# Broad-spectrum monoclonal antibodies against chikungunya virus structural proteins: Promising candidates for antibody-based rapid diagnostic test development

**DOI:** 10.1371/journal.pone.0208851

**Published:** 2018-12-17

**Authors:** Aekkachai Tuekprakhon, Orapim Puiprom, Tadahiro Sasaki, Johan Michiels, Koen Bartholomeeusen, Emi E. Nakayama, Michael K. Meno, Juthamas Phadungsombat, Ralph Huits, Kevin K. Ariën, Natthanej Luplertlop, Tatsuo Shioda, Pornsawan Leaungwutiwong

**Affiliations:** 1 Department of Microbiology and Immunology, Faculty of Tropical Medicine, Mahidol University, Bangkok, Thailand; 2 Mahidol-Osaka Center for Infectious Diseases (MOCID), Faculty of Tropical Medicine, Mahidol University, Bangkok, Thailand; 3 Department of Biomedical Sciences, Institute of Tropical Medicine, Antwerp, Belgium; 4 Research Institute for Microbial Diseases (RIMD), Osaka University, Osaka, Japan; 5 Department of Clinical Sciences, Institute of Tropical Medicine, Antwerp, Belgium; 6 Department of Biomedical Sciences, University of Antwerp, Antwerp, Belgium; Faculty of Science, Ain Shams University (ASU), EGYPT

## Abstract

In response to the aggressive global spread of the mosquito-borne chikungunya virus (CHIKV), an accurate and accessible diagnostic tool is of high importance. CHIKV, an arthritogenic alphavirus, comprises three genotypes: East/Central/South African (ECSA), West African (WA), and Asian. A previous rapid immunochromatographic (IC) test detecting CHIKV E1 protein showed promising performance for detection of the ECSA genotype. Unfortunately, this kit exhibited lower capacity for detection of the Asian genotype, currently in circulation in the Americas, reflecting the low avidity of one of the monoclonal antibodies (mAbs) in this IC kit for the E1 protein of the Asian-genotype because of a variant amino acid sequence. To address this shortcoming, we set out to generate a new panel of broad-spectrum mouse anti-CHIKV mAbs using hybridoma technology. We report here the successful generation of mouse anti-CHIKV mAbs targeting CHIKV E1 and capsid proteins. These mAbs possessed broad reactivity to all three CHIKV genotypes, while most of the mAbs lacked cross-reactivity towards Sindbis, dengue, and Zika viruses. Two of the mAbs also lacked cross-reactivity towards other alphaviruses, including O'nyong-nyong, Ross River, Mayaro, Western Equine Encephalitis, Eastern Equine Encephalitis, and Venezuelan Equine Encephalitis viruses. In addition, another two mAbs cross-reacted weakly only with most closely related O'nyong-nyong virus. Effective diagnosis is one of the keys to disease control but to date, no antibody-based rapid IC platform for CHIKV is commercially available. Thus, the application of the mAbs characterized here in the rapid diagnostic IC kit for CHIKV detection is expected to be of great value for clinical diagnosis and surveillance purposes.

## Introduction

Chikungunya virus (CHIKV), an arthritogenic mosquito-borne virus, was first documented more than six decades ago in Tanzania, East Africa [1]. Since then, CHIKV has caused sporadic outbreaks throughout the African and Asian continents. Although only one serotype of CHIKV exists, the virus is classified into three genotypes named after the geographical location where the respective genotype was first recognized: East/Central/South/African (ECSA), West African (WA), and Asian [2]. CHIKV was considered a neglected tropical agent until a massive outbreak was reported on islands of the Indian Ocean in 2005. The outbreak affected one-third of the population in this region and caused more than 200 mortalities [3, 4]. Although the causative agent was shown to originate from the ECSA genotype, in-depth analysis confirmed that this virus showed characteristic genomic microevolution, and so isolates of this clade were designated as ECSA genotype Indian Ocean Lineage (ECSA-IOL) [4, 5]. Following its initial detection in the Indian Ocean islands, ECSA-IOL also was identified as the cause of several CHIKV outbreaks in India and South East Asia (including Thailand, Cambodia, and Malaysia), and ECSA-IOL was introduced for the first time into European countries (including Italy and France) [6, 7]. Additionally, Asian-genotype CHIKV subsequently gave rise to a novel clade, designated as Asian/American [8]. This clade now is considered a public health concern in several countries/territories in the Caribbean and in the Central American mainland [9, 10]. Approximately one billion people are estimated to live in areas where CHIKV is in circulation [7]. Thus, despite originally being considered a tropical infectious agent, CHIKV is now considered a global health challenge. As of 2018, there is no specific treatment for chikungunya disease, and a chikungunya vaccine is still under development [11]. Given these limitations in prevention and control, it is highly likely that CHIKV (along with its insect vector) will continue to spread, increasing the risk of CHIKV infection world-wide.

CHIKV (genus *Alphavirus*; family *Togaviridae*) is an enveloped virus that possesses a positive-sense, single-stranded RNA genome. This genome is approximately 11.8 kb in length, although this size has been found to vary slightly among isolates [12]. Like other alphaviruses, CHIKV has two open reading frames (ORFs). The 5' ORF encodes four non-structural proteins (nsP1-nsP2-nsP3-nsP4) that provide important enzymatic activities during viral replication [13]. The 3' ORF, which is expressed from a subgenomic RNA, encodes structural polyproteins, comprising the capsid (C) and envelope (E3-E2-6K-E1) proteins [12]. During viral assembly, the 30-kDa C protein is needed for the packaging of newly synthesized genomic RNAs. The envelope proteins E2 and E1, which are incorporated as trimeric spikes on the mature virion, are responsible for receptor binding and fusion with host membrane protein(s) [14, 15]. The smaller envelope proteins, E3 (64 amino acid (aa) residues) and 6K (66 aa residues), are not primary components of the mature virion; instead, these proteins act together as a signal sequence facilitating translocation of the precursor envelope protein to the membrane of the endoplasmic reticulum (ER) [16, 17]

CHIKV E1 protein consists of approximately 439 aa residues (CHIKV reference strain, S27 strain, Accession Number NC004162.2). CHIKV E1 protein is a type-I transmembrane protein comprising three domains: DI, DII, and DIII. The central domain, DI, contains two long insertions that form DII, a domain that includes a hydrophobic fusion loop. DIII connects to DI via a linker region and to a transmembrane region that attaches the co-domain of the E1 protein to the viral membrane [18, 19]. E2-E1 heterodimers form on the surface of the intact virion, such that DII of the CHIKV E1 protein lies laterally alongside E2, with the fusion loop of E1 buried in the groove-forming domains A and B of E2 [20]. These arrangements are important not only for CHIKV attachment and the membrane fusion process, but also for the folding and transport of the newly produced E1 protein to the plasma membrane of CHIKV-infected cells [21].

Both the structural and non-structural proteins of CHIKV have been reported to contain B-cell epitopes. However, E2 and (to a lesser extent) nsP3 contain the epitopes most frequently targeted by antibodies from infected individuals [22]. Although CHIKV E1 is located on the viral surface, there are few examples of neutralizing antibodies that target E1 [22, 23]. Studies of alphaviruses have indicated that the recognition of the E1 protein by monoclonal antibodies (mAbs) depends on several conditions, including the dissociation of the E2-E1 heterodimer [24, 25] or the exposure of virions to low pH [26]. To date, mouse anti-CHIKV E1 antibodies have been generated and employed primarily in the context of immunodiagnostic test development [23, 27, 28].

More than 75% of CHIKV-infected individuals develop a classical acute clinical presentation [29] that is similar to that observed in patients infected by other mosquito-borne viruses such as dengue and Zika virus [30]. These symptoms present diagnostic challenges to clinicians working in geographic areas where these viruses cocirculate [31]. Although debilitating joint pain is a distinctive feature of CHIKV infection, many of the other clinical symptoms and pathology of CHIKV infection are not specific to CHIKV and may occasionally become severe. Accurate diagnosis of CHIKV is thus clinically relevant for supportive management of the infected patient, and to avoid unnecessary diagnostic procedures.

To diagnose CHIKV infection in the acute (viremic) phase, several polymerase chain reaction (PCR) -based methods and a point-of-care immunochromatographic (IC) device that detects CHIKV E1 protein have been described [28]. Given that the IC test is user-friendly, cost-effective, and facilitates early diagnosis, this device was expected to increase accessibility of laboratory diagnosis of CHIKV infection. Indeed, this IC device has been reported to exhibit high sensitivity (89.4%) and specificity (94.4%) against clinical samples known to contain ECSA-genotype CHIKV [28]. However, a further evaluation of this device with confirmed clinical samples of Asian-genotype CHIKV revealed low (33.3%) sensitivity towards virus of this genotype [32]. In further investigation, we showed that one of the mAbs used in this test has a limited ability to recognize the Asian-genotype 6K-E1 protein, which differs from the ECSA-genotype 6K-E1 protein as the result of a glutamic acid (E) to aspartic acid (D) substitution at aa 350 [33]. This mismatch is crucial, since these two genotypes remain the major causes of chikungunya epidemics worldwide [8].

Here, we describe the successful generation and characterization of superior anti-CHIKV mAbs targeting the E1 and C proteins of CHIKV. These mAbs were shown to exhibit broad reactivity towards all genotypes currently in circulation around the globe. Given these promising properties, these new mAbs might be further applied to the development of an improved CHIKV antigen-detection diagnostic test.

## Materials and methods

### Ethics statement

All procedures in the animal studies described here were carried out in accordance with the recommendations of “The Guide for the Care and Use of Laboratory Animals” of Osaka University, Japan. All animal experiments were approved by the Animal Care and Use Committee of the Research Institute for Microbial Diseases, Osaka University, Japan (Approval Number H27-10-1). All procedures were performed using best efforts to minimize animal suffering.

### CHIKV-specific mouse mAbs

CHIKV-specific mouse mAbs CK47 and CK119 [34] were used as control antibodies for the detection of CHIKV envelope protein. The anti-CHIKV capsid protein mouse mAb, 19B02 (The Native Antigen Company, Oxford, United Kingdom), was used as a positive control for detecting the expression of CHIKV capsid protein. Anti-flavivirus mAb 4G2 (hybridoma D1-4G2-4-15; ATCC HB-112) was used as a positive control for flavivirus detection.

### Cells and viruses

African green monkey kidney epithelium-derived cells (Vero cells; ATCC CCL-81), glioblastoma-derived human cell line U251 (JCRB IFO50288), and BALB/c mouse-derived B7 cells [35] were maintained in Minimum Essential Medium (MEM; Life Technologies, Inc., USA). The Human Embryonic Kidney cell line (HEK293T; ATCC CRL-3216) and the Baby Hamster Kidney fibroblast cell line (BHK; ATCC CCL-10) were maintained in Dulbecco’s Modified Eagle’s Medium (DMEM; Life Technologies). The Chinese Hamster Ovary (CHO) cell line was kindly provided by Dr. Makoto Takeda at the National Institute of Infectious Disease in Japan and was maintained in RPMI medium. PAI, a mouse myeloma cell line [36], was maintained in RPMI medium. For normal cultivation conditions, the base medium was supplemented to 10% (v/v) with heat-inactivated Fetal Bovine Serum (FBS; Life Technologies); for cultivation of infected cells, the base medium was supplemented only to 2% FBS. All cells were maintained at 37°C in a 5% CO_2_ environment. *Aedes albopictus*-derived cells (C6/36) were cultivated in Leibovitz’s L-15 medium (Hyclone; GE Healthcare, Uppsala, Sweden) supplemented to 10% FBS and 0.3% tryptose phosphate broth (TPB) (Becton Dickinson, Franklin, NJ).

CHIKV CP10 and Ross Low psg strains were used as representatives of the ECSA genotype, while ARUBA-1125 [32] was used as a representative of the Asian genotype. One alphavirus, Sindbis virus (SINV; R68 strain), was kindly provided by Dr. Kohji Moriishi, University of Yamanashi. Alphaviruses O’nyong’nyong virus (ONNV, strain Ahero), Mayaro virus (MAYV, strain TC652), Ross River virus (RRV, strain 0005281v), Venezuelan Equine Encephalitis virus (VEEV, strains 78v and TrD), Eastern Equine Encephalitis virus (EEEV, strain H178/99), SINV (strain EGAR339), and Western Equine Encephalitic virus (WEEV, strain H160/99) were purchased from National Collection of Pathogenic Viruses (Public Health England, UK). Four serotypes of dengue virus (DENV; using strains Mochizuki, 16681, H87, and H241 as representatives of the DENV1 to DENV4 serotypes, respectively) were used to assess cross-reactivity. We propagated CHIKV, SINV, and DENV in Vero, BHK, and C6/36 cells, respectively.

### Expression vectors for CHIKV envelope and capsid proteins

To screen and determine the reactivity of anti-CHIKV mAbs, plasmids encoding CHIKV envelope and capsid proteins were employed as described previously [33]. Briefly, a codon-optimized complementary DNA (cDNA) encoding CHIKV C protein (37997 strain; NCBI Accession No. AY726732.1) and the envelope protein for each of the three genotypes of CHIKV (ECSA: CP10 strain, NCBI Accession No. AB857817.1; WA: 37997 strain, NCBI Accession No. AY726732.1; and Asian: CK12-686 strain, NCBI Accession No. CWIH01000001.1) were commercially synthesized (GenScript, Piscataway, NJ). Notably, the synthetic cDNAs for the envelope protein genes of CP10 and 37997 strains were designed to encode the proteins with HA tags at their C-termini. To express the C or envelope proteins (expressed either as E3-E2-6K-E1, 6K-E1, or 6K), these cassette cDNAs were inserted (individually) into the expression plasmid pCAGGS MCSII [37]. To generate a plasmid encoding E1 without 6K, we inserted the sequence encoding HA-tagged E1 into pFUSE2ss-CLIg-mk (an IL2 signal sequence-providing plasmid) using flanking EcoRI and NheI sites (Invivogen, USA). To study the reactivity of mAbs towards envelope proteins harboring an amino acid substitution, we used overlapping primers (as described previously [38]) to introduce mutations into the DNA sequence encoding amino acid 350 of the CHIKV 6K-E1 protein. Specifically, a mutant version of the CP10 gene was generated to encode a glutamic acid-to-aspartic acid substitution (E350D), while mutant versions of the 37997 and CK12-686 genes were generated to encode an aspartic acid-to-glutamic acid substitution (D350E).

### Pseudotyped lentiviral vector for expression of CHIKV envelope proteins

To increase the efficacy, safety, and convenience of the neutralization assay, a well-established pseudotyped lentiviral system was employed. The construction of CHIKV-pseudotyped lentiviral vector was performed as described previously by Kishishita *et al*. [37], with some modifications. Briefly, a mixture containing three plasmids [pCAGGS MCSII containing a cDNA encoding CHIKV Asian-genotype CK12-686 (2.6 μg); pLenti CMV Puro LUC (w168-1), which carries a firefly *luciferase* reporter gene (9 μg); and psPAX2, a lentivirus packaging vector (9 μg)] was prepared in one milliliter of serum-free DMEM medium. The plasmids were co-transfected into HEK293T cells using a nucleic acid delivery reagent, polyethylenimine (PEI) (Polysciences, Inc.; Warrington, PA). The resulting transfected HEK293T cells were maintained in DMEM supplemented with 10% FBS for another 48 hours. Culture supernatant containing CHIKV envelope protein-pseudotyped lentivirus was harvested, and the viral titer was determined by measuring the level of HIV-1 gag p24 protein using the RETROtek HIV-1 p24 antigen ELISA kit (ZeptoMetrix; Buffalo, NY), according to the manufacturer’s instructions.

### Sendai virus (SeV) for expression of CHIKV 6K-E1 envelope protein

The construction of a recombinant SeV expressing foreign genes was performed as described previously [39, 40] with some modifications. Briefly, the 6K-E1-encoding codon-optimized gene of CHIKV Asian-genotype strain CK12-686 was inserted into the parental pSeV18b(+) plasmid [39] at the NotI site, yielding a construct capable of generating a SeV expressing the Asian-genotype CHIKV 6K-E1 protein. The resulting construct (5 μg) and plasmids encoding viral nucleocapsid (N) and RNA polymerase (P and L) trans-acting proteins (pGEM-N, 2 μg; pGEM-P, 1 μg; and pGEM-L, 2 μg, respectively) were cotransfected into a Chinese Hamster Ovary cell line (CHO) infected with a vaccinia virus expressing T7 RNA polymerase [41]. The resulting cells were maintained in RPMI containing 10% FBS for another 72 hours. SeV expressing CHIKV 6K-E1 envelope protein then was propagated in embryonated chicken eggs for another two passages and used for further experiments.

### Immunogen design and preparation

We prepared two different kinds of immunogens: B7 cells infected with ECSA-genotype CHIKV and those infected with SeV expressing the Asian-genotype CHIKV 6K-E1 protein. Briefly, confluent B7 cells were seeded in 10-cm culture dishes at approximately 6.5 x 10^6^ cells/dish and grown overnight. ECSA-genotype CHIKV (at a multiplicity of infection (MOI) of 0.1) or SeV expressing the Asian-genotype CHIKV 6K-E1 (at a MOI of 10) was inoculated to the B7 cells for two days or until cell-infection rates exceeding 90% were observed. Infected cells were collected and washed by centrifugation (1000 rpm, 5 min) with sterile phosphate-buffered saline (PBS). After the supernatant was discarded, the cells were resuspended in 10 mL of 0.4% (v/v) paraformaldehyde and the suspension was incubated at 4°C for 48 hours. Paraformaldehyde-treated cells then were collected by centrifugation and washed twice with PBS. After the final washing, the infected B7 cells were resuspended at 2.5 x 10^7^ cells/mL in PBS and held at -80°C pending use in mouse immunization.

### Immunization strategies and mAb production

Mouse mAbs against CHIKV were generated according to the procedure described previously [27] with some modifications, Briefly, three mice (BALB/c CrSIc, 4 weeks, female, specific pathogen free, 15–20 g each; Japan SLC, Inc.) were used for immunization. Each mouse was housed individually in a plastic mouse cage (KN-600, Natsume Seisakusho Co., Ltd.) with paper chips (Paper clean, Japan SLC, Inc.) and free access to rodent diet (CE-2, CLEA Japan Inc.) and water in a standard laboratory environment. Body weights were recorded once weekly; no abnormalities were observed during the in-life interval. Three distinct immunization strategies (A, B, and C) were employed in each of the three mice (Table 1 and S1 Fig). One hundred microliters of B7 cells (total 2.5 x 10^6^ cells) infected with ECSA-genotype CHIKV or SeV expressing the Asian-genotype CHIKV 6K-E1 were mixed with an equal volume of complete (for priming) or incomplete (for boosting) Freund’s adjuvant (Sigma-Aldrich, Saint Louis, MO). In all three strategies, mice were prime-immunized intraperitoneally with ECSA-genotype-infected B7 cells and boosted at two-week intervals. In strategy A, the mouse was boosted twice with B7 cells infected with SeV expressing the Asian-genotype CHIKV 6K-E1. In strategy B, the mouse was boosted once with ECSA-infected B7 cells, followed by B7 cells infected with SeV expressing the CHIKV Asian-genotype 6K-E1. In strategy C, the mouse was boosted twice with CHIKV ECSA-infected B7 cells. Three days after the final booster, the mice were euthanized by cervical dislocation performed by a well-trained personnel and the spleens were harvested and processed. Hybridomas were generated by fusing splenocytes from individual mice with PAI fusion partner cells, a process facilitated by polyethylene glycol (PEG) 1500 (Roche Applied Sciences, Germany). To select hybridomas, fused cells were seeded in 96-well plates and maintained in DMEM supplemented with 15% FBS and hypoxanthine-aminopterin-thymidine (HAT) (Invitrogen, Carlsbad, CA) and hypoxanthine-thymidine (HT) (Invitrogen). The culture medium was changed every three days for two weeks. Culture supernatants from successfully fused cells were subjected to primary screening, using an indirect immunofluorescence test (IIFT), to identify hybridomas secreting CHIKV-specific immunoglobulin (Ig). Cells positive in the primary IIFT screen then were subjected to limiting dilution to isolate single parental cells that produced mAbs. All selected parental cells were subjected to a second round of screening prior to being scaled up.

**Table 1:** Summary of mouse immunization and screening of hybridoma clones.

	Immunogen			Fusion	No. of clones after screening		
Strategy	Prime ^a^	1st boost ^b^	2nd boost ^b^	efficiency ^e^(%)	1^st^	2^nd^	3^rd^
A	ECSA/CHIKV^c^	Asian/SeV^d^	Asian/SeV^d^	87.7	32	0	0
B	ECSA/CHIKV^c^	ECSA/CHIKV^c^	Asian/SeV^d^	86.9	304	34	9
C	ECSA/CHIKV^c^	ECSA/CHIKV^c^	ECSA/CHIKV^c^	62.9	336	17	6

^a^: Freund’s complete adjuvant

^b^: Freund’s incomplete adjuvant

^c^: B7 cells infected with chikungunya virus

^d^: B7 cells infected with recombinant Sendai virus

^e^: Percentage of hybridoma colony positive wells

### Indirect immunofluorescence test (IIFT)

To determine reactivity and cross-reactivity of mAbs, we performed IIFT according to the procedure described previously [27]. To test reactivity, we prepared 96-well plates containing Vero cells infected with CHIKV or HEK293T cells transfected with plasmids encoding CHIKV envelope or C proteins. To test cross-reactivity, we prepared 96-well plates containing one of the following: BHK cells infected with SINV virus, or Vero cells infected with ONNV, MAYV, RRV, VEEV, EEEV, SINV, or WEEV. We also prepared 96-well plates containing Vero cells infected with each of the four serotypes of DENV or with Zika virus (ZIKV) (SV0010/15; kindly provided by Akanitt Jittmittraphap, Department of Microbiology and Immunology, Faculty of Tropical Medicine, Mahidol University). After an 80% cytopathic effect was observed (in viral infection) or at 48 hours post-transfection, infected or transfected cells were washed three times with PBS, fixed with formaldehyde (3.7% (v/v) in PBS), and permeabilized with Triton X-100 (1% (v/v) in water). After another 3 rounds of washing with PBS, 50 μL of primary antibody (culture supernatant for screening, or purified anti-CHIKV mAbs (2 μg/mL) for characterization) was applied to the infected or transfected cells and the plates were incubated at 37°C for 1 hour. The liquid was removed by aspiration and PBS was added to each well three times to ensure that the unbound antibody was removed. Fifty microliters of the secondary antibody (green-fluorescent dye (Alexa Fluor 488; Invitrogen) -conjugated goat-anti mouse IgG) was dispensed into each well and the plates were incubated for 45 minutes at 37°C, followed by another washing step. To detect HA-tagged protein in plasmid-transfected cells, cells were incubated with 50 μL of rat anti-HA peptide mAb (0.5 μg/mL high-affinity anti-HA antibody; Roche Applied Sciences) for 1 hour, followed by incubation with red-fluorescent dye (Alexa Fluor 555; Invitrogen) -conjugated goat-anti rat IgG for another hour. To visualize cell nuclei, 50 μL of 4’,6-diamidino-2-phenylindole dihydrochloride (DAPI) (Sigma-Aldrich) (1 μg/mL in PBS) was dispensed into each well. The reactivity of the anti-CHIKV antibody, reactivity of the anti-HA antibody, and presence of nuclei, indicated by green (excitation/emission at 488 and 525 nm, respectively), red (555 nm/568 nm), and blue (360 nm/460 nm) fluorescence, respectively, were observed using a fluorescence microscope (IX71, Olympus, Tokyo, Japan or Carl Zeiss, Oberkochen, Germany, or ECLIPSE Ti2, Nikon, Tokyo, Japan). Images from IIFT experiments with multiple fluorescent colors were overlaid using ImageJ 1.5Oi (National Institutes of Health USA).

### Monoclonal antibody isotype determination

To determine the isotype of the heavy and light chains of the Igs generated by the mAb-producing hybridomas, individual clones were tested with a commercially available mouse mAb isotyping kit (Isostrip; Roche Applied Sciences) according to the manufacturer’s instructions.

### Western blotting analysis

To determine the linear epitopes recognized by the mAbs, western blot analysis was performed. CHIKV- or mock-infected cell lysates were mixed with 2× Laemmli sample buffer under reducing or non-reducing conditions (with or without β-mercaptoethanol, respectively) and heated at 95°C for 5 min. Prepared samples and pre-stained protein marker (10–250 kDa, Kaleidoscope; Bio-Rad) were separated in a 4–12% SDS-PAGE gradient gel (NuPAGE, Carlsbad, CA) and transferred onto methanol-pretreated polyvinylidene fluoride (PVDF) membranes (Bio-Rad). To prevent non-specific binding, membranes with transferred proteins then were incubated with 5% (w/v) skim milk in Tris-buffered saline with 0.05% Tween 20 (TBST) for 1 hour, with gentle agitation, at room temperature. The membrane then was cut into small slices and reacted with individual anti-CHIKV mAbs (culture supernatant (1:2 v/v) or purified mAb (2 μg/mL)) in blocking buffer, overnight at 4°C. Subsequently, individual slices were washed three times (5 min each) with TBST and then incubated for 1 hour in the presence of horseradish peroxidase-conjugated goat anti-mouse IgG (KPL antibody; SeraCare Life Science, Inc., Mildford, MA), diluted 1:10,000 (v/v) in blocking buffer. After another three washes with TBST, the reactivity of mAbs to CHIKV protein was detected by chemiluminescent substrate (Amersham ECL western blot; GE Healthcare) and imaged using a charge-coupled device (CCD) imager (ImmageQuant LAS 500; GE Healthcare).

Besides CHIKV-infected cells lysates, our mAbs also were tested against two other types of antigen: plasmid-transfected cells lysates and viral (CHIKV) particles. To generate plasmid-transfected cell lysates, plasmid encoding CHIKV C protein was prepared. Two hundred and fifty microliters of a mixture of plasmid (2 μg) and PEI nucleic acid delivery reagent (Polysciences, Inc.) were transfected into each well of a 6-well plate containing HEK293T cells. At 72 hours post-transfection, cells were lysed using NP-40 lysis buffer. Viral particles were isolated from the culture medium by ultracentrifugation at 50,000 x g for 2.5 hours (Optima MAX-XP; Beckman-Coulter, IN, USA) and viral titers were adjusted to 2 x 10^5^ plaque forming units (PFU) per mL with PBS. Subsequent western blotting analyses with the cell lysates and particles were performed as described above.

### Neutralization assay

To determine the neutralizing activity of anti-CHIKV envelope proteins, a system of pseudotyped lentiviral particles harboring a *luciferase* reporter gene and CHIKV envelope proteins was employed in a neutralization test [33, 37]. Individual anti-CHIKV E1 mAbs (0.7 mg/mL) were subjected to a four-fold serial dilution, yielding mAb at concentrations ranging from 1:200 to 1:204800. Pseudotyped lentivirus bearing Asian-genotype CHIKV envelope proteins were pre-incubated for 2 hours with each set of mAb dilutions prior to infection of U251 cells. Infected cells were maintained in MEM supplemented with 2% (v/v) FBS for another 72 hours. Finally, culture medium was replaced with 100 μL of PBS and an equal volume of luciferase assay reagent (Bright-Glo Luciferase assay system; Promega, Madison, WI, USA) was added prior to quantifying luminescence using a Synergy H1 microplate reader with Gen5 software (Biotek Winooski, VT, USA). Neutralizing activity of anti-CHIKV envelope protein against the pseudotyped lentivirus was determined as the percentage of infection of pseudotyped lentivirus, calculated using the following equation: (relative luciferase units (RLUs) of cells infected with pseudotyped lentivirus and mAb mixture divided by RLUs of cells infected with pseudotyped lentivirus alone) x 100%.

### Amino acid sequence analysis of CHIKV capsid protein

To determine amino acid variation of the CHIKV C protein among genotypes, we retrieved representative CHIKV capsid protein sequences from the NCBI database. For CHIKV Asian-genotype strain ARUBA-1125, the amino acid sequence was deduced from the nucleotide sequence of the viral genome. A double-stranded complementary DNA (cDNA) was generated from the viral RNA of a low-passage isolate of CHIKV strain ARUBA-1125 using primer pCMV-CHIK-R (TGCCATGCCGACCCTGAACATTA) for reverse transcription and primer pair CHIKV-5605F (AGCGACTGGTCCACGTGCT) and CHIK-R-long (GAAATATTAAAAACAAAATAACATCTCCTACGTCCCTGT) for PCR-amplification. The nucleotide sequence of the C protein gene in the amplified fragment was determined using primers 7449F (TCAGAGGACCCGTCATAACC), 7817F (CCAAAAAGAAACCGGTTCAA), and 8435R (TTCTCCGGCTCTTTTTCGTA). The capsid protein-encoding DNA sequence then was translated to yield the amino acid sequence. Multiple sequence alignment was performed to compare amino acid variations among representative strains from all three genotypes using Molecular Evolutionary Genetic Analysis (MEGA) software, version 7.0 [42]

## Results

### Expression of CHIKV 6K-E1 proteins by recombinant SeV

**Fig 1:**
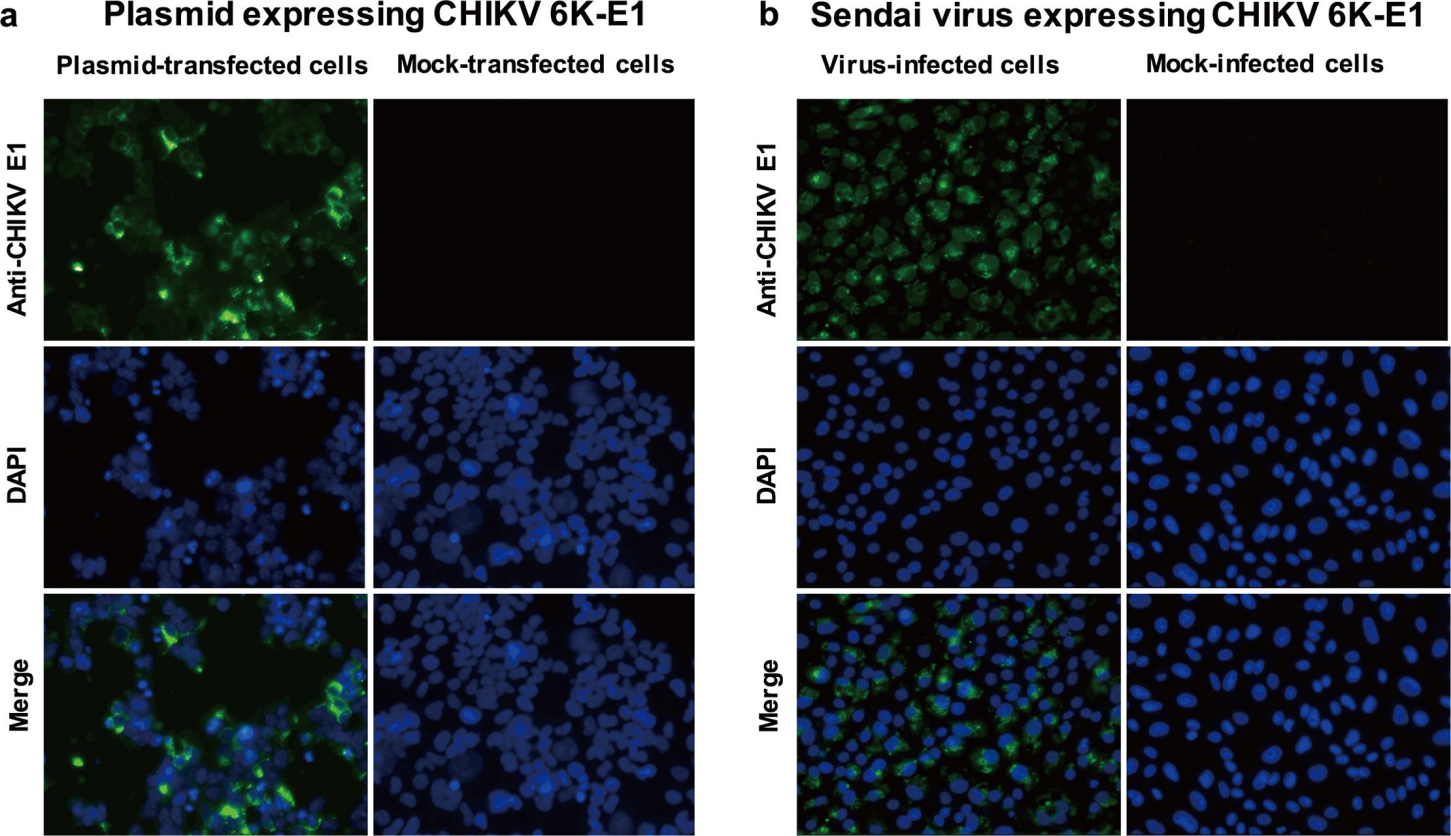
Expression of CHIKV 6K-E1 proteins by recombinant Sendai virus. Indirect immunofluorescent test was performed using anti-CHIKV E1 (monoclonal antibody CK119); labeling was detected with Alexa Fluor 488-conjugated secondary antibody (green color). DAPI nuclear counterstain was used to stain nuclei of cells (blue color). (a) HEK293T cells transfected with plasmid encoding CHIKV Asian-genotype 6K-E1 protein (pCEP4/CK12-686 6K-E1) or mock-transfected. (b) Vero cells infected with Sendai virus expressing the Asian-genotype CHIKV 6K-E1 protein or mock-infected. Images represent two independent experiments with similar results, taken under 40x objective magnification using an Axio observer Z.1 microscope (Carl Zeiss). Merged images of anti-CHIKV E1 and DAPI (Merge) also are shown.

The SeV vector is a powerful tool for gene delivery/expression and has been used to express a number of exogenous genes [40]. In the study presented here, we employed the SeV vector for expression of CHIKV envelope 6K-E1 proteins for use in mouse immunization. Prior to production of recombinant SeV encoding CHIKV 6K-E1, a synthetic 6K-E1-encoding region from an Asian-genotype CHIKV was subcloned into the pCEP4 expression plasmid. The expression of 6K-E1 protein from the resulting plasmid (pCEP4/CK12-686 6K-E1) was confirmed by a positive reaction in IIFT using an anti-CHIKV E1 protein antibody (mAb CK119) [34] (Fig 1A) prior to incorporation into the SeV expression system. The observation that Vero cells infected with SeV expressing the Asian-genotype CHIKV 6K-E1 reacted positively with CK119 in IIFT (Fig 1B) confirmed that the SeV system was able to express the CHIKV 6K-E1 envelope protein, which therefore could be used for further immunization.

### Production of mAbs against CHIKV

Three immunization strategies (designated A, B, and C) were employed using each of 3 mice (Table 1 and S1 Fig). The fusion efficiency between splenocytes and PAI cells was assessed as the ability of cells to grow in the HAT selective medium. We observed fusion efficiencies of more than 50% in all three strategies (Table 1). In the primary screen to identify anti-CHIKV Ig-positive clones, culture supernatants from clones generated from strategies A and B were tested by IIFT for reactivity against HEK293T cells transfected with a plasmid encoding the Asian-genotype 6K-E1 protein (pCEP4/CK12-686 6K-E1). Supernatants of clones obtained by strategy C were screened for reactivity against CHIKV (CP10 strain) -infected Vero cells. Immunization strategy C gave the highest number of CHIKV-specific Ig clones (21 clones), followed by B (19 clones) and A (2 clones) (Table 1). Compared to virus-infected cells, plasmid-transfected cells had lower levels of antigen expression. Since we screened clones from immunization strategy B with plasmid-transfected cells, we may have missed clones expressing antibodies with relatively low affinity for CHIKV.

To obtain single antibody-producing cells, we performed limiting dilution on all positive clones. From each, 16 subclones were subjected to secondary screening by IIFT with the same antigen as that used in the primary screen. Secondary screening of the 32 strategy-A clones (derived from the 2 clones generated by strategy A) failed to detect a single positive hybridoma. On the other hand, secondary screening of the clones obtained from strategies B and C yielded 11.18% (34/304) and 5.04% (17/337) positive clones, respectively (Table 1).

To ensure that all positive clones retained the function of antibody production during the scaling-up process, culture supernatants of clones were tested with the same antigen as that used in the primary and secondary screenings. For clones derived from strategy B, 35.3% (12/34) displayed strong reactivity against cells transfected with a plasmid encoding Asian-genotype 6K-E1 protein. None of the clones reacted with mock-transfected HEK293T cells, but 17.6% (6/34) showed cross-reactivity with SINV. For clones derived from strategy C, 64.7% (11/17) exhibited strong reactivity against cells infected with the CP10 virus; one clone (5.9%, 1/17) reacted with uninfected Vero cells, and 64.7% (11/17) displayed cross-reactivity to SINV. For further characterization, we selected nine and six clones derived from immunization strategies B and C, respectively, based on their strength of reactivity and specificity (the 3^rd^ screening in Table 1 and S1 Fig).

### Isotype profiles and reactivity towards CHIKV-infected Vero cells of anti-CHIKV mAbs

**Fig 2:**
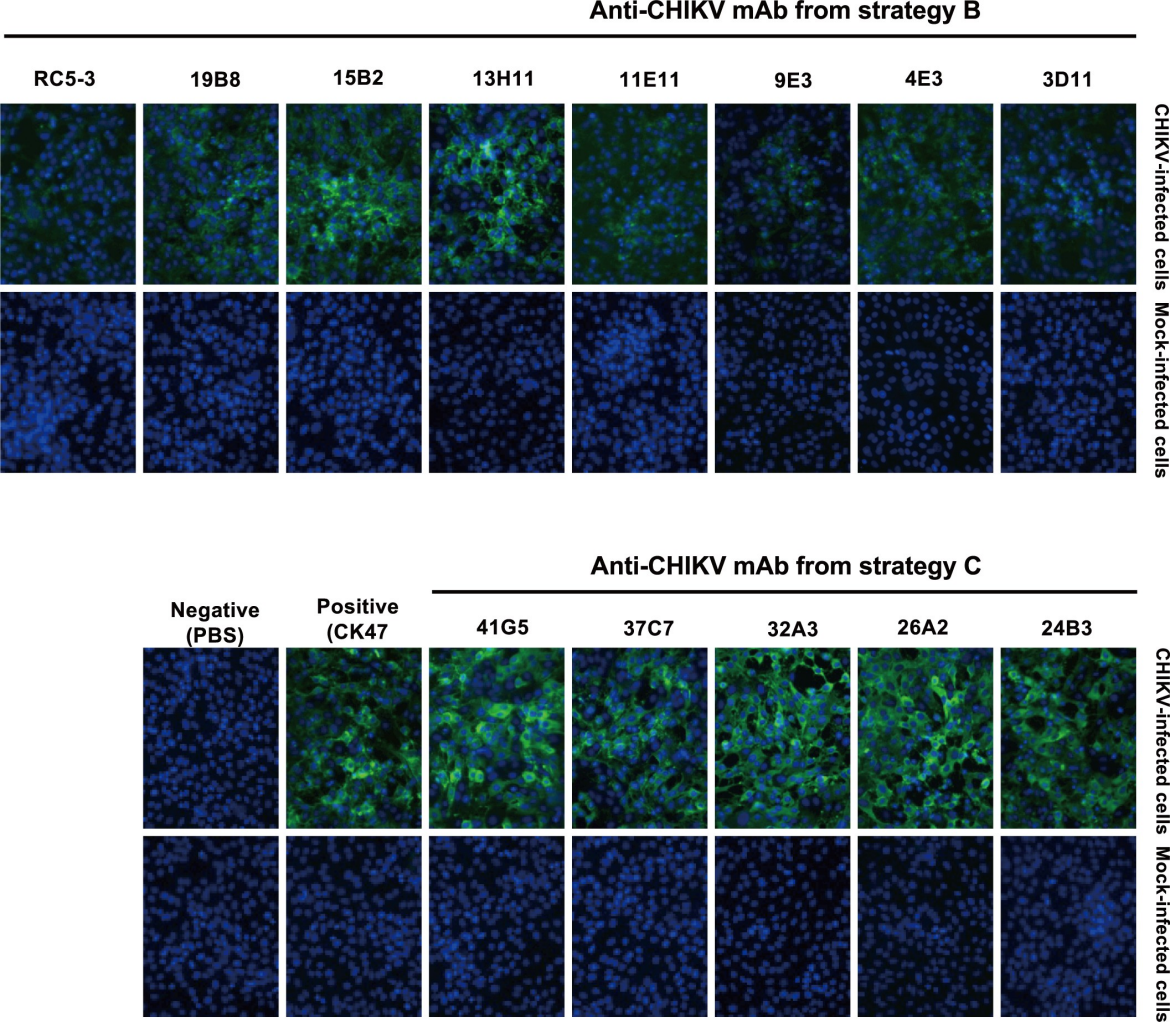
Reactivity of anti-CHIKV monoclonal antibodies (mAbs). Indirect immunofluorescent test was performed against CHIKV (ECSA, CP10 strain) -infected Vero cells using anti-CHIKV mAbs generated by strategies B (left) and C (right) in the present study. The reactivity of each mAb was detected by Alexa Fluor 488-conjugated secondary antibody (green color), while DAPI nuclear counterstain was used to stain nuclei of cells (blue color, right-hand panels). Anti-CHIKV E1 mAb (CK47) was used as the control antibody for CHIKV infection, while the antibody diluent (PBS) was used as the negative control. Images are representative of results obtained from three independent experiments with similar results and were taken under 40x objective magnification using an inverted microscope (Axio observer Z.1, Carl Zeiss). The images of Alexa Fluor 488 and DAPI staining were merged using ImageJ 1.5Oi, National Institutes of Health USA (left-hand panels).

The fifteen selected clones derived from strategies B and C were subjected to Ig subtyping. As detailed in Table 2, the isotype profile of Ig heavy and light chains was performed using a commercial isotyping kit. Among the 15 selected clones, 66.7% (10 clones) were IgG1, 13.3% (2 clones) were IgG2a, and 6.7% (1 clone) each were IgG2b, IgM, and IgA. All of the clones produced kappa (κ) light chains. We excluded 6B6 (IgA) and 35B7 (IgM) from the subsequent analyses since our aim was to obtain IgG antibodies against CHIKV. The reactivities of the remaining 13 antibody clones towards CHIKV-infected Vero cells are shown in Fig 2. Although the binding affinity of individual mAbs towards CHIKV-infected cells varied among clones, all 13 clones showed positive reactions. There was a tendency for the antibodies obtained from strategy C (24B3, 26A2, 32A3, 37C7, and 41G5) to show stronger staining than did the antibodies obtained from strategy B (3D11, 4E3, 9E3, 11E11, 13H11, 15B2, 19B8, and RC5-3).

**Table 2:** Characteristics of anti-CHIKV mAbs.

Strategy	mAb	Isotype H, L	Western blot analysis^a^		Binding region
			reducing	non-reducing	
**B**	**3D11**	IgG2a, κ	non-reactive	non-reactive	E1
**B**	**4E3**	IgG1, κ	non-reactive	non-reactive	ND
**B**	**6B6**	IgA, κ	ND	ND	ND
**B**	**9E3**	IgG1, κ	non-reactive	non-reactive	ND
**B**	**11E11**	IgG1, κ	non-reactive	48 kDa	E1
**B**	**13H11**	IgG1, κ	non-reactive	non-reactive	E1
**B**	**15B2**	IgG2b, κ	non-specific	non-specific	E1
**B**	**19B8**	IgG2a, κ	non-reactive	non-reactive	E1
**B**	**RC5-3**	IgG1, κ	non-reactive	non-reactive	E1
**C**	**24B3**	IgG1, κ	35 kDa	35 kDa	Capsid
**C**	**26A2**	IgG1, κ	35 kDa	35 kDa	Capsid
**C**	**32A3**	IgG1, κ	35 kDa	35 kDa	Capsid
**C**	**35B9**	IgM, κ	ND	ND	ND
**C**	**37C7**	IgG1, κ	35 kDa	35 kDa	Capsid
**C**	**41G5**	IgG1, κ	35 kDa	35 kDa	Capsid
**Control** **mAb**	**CK47**	IgG2a, ND	50 kDa	48 kDa	E1

Note; H: Heavy chain; L: Light chain, ND: Not determined.

^a^ Monoclonal antibody-containing culture supernatant versus CHIKV-infected cell lysate.

### Immunoblot analysis

**Fig 3:**
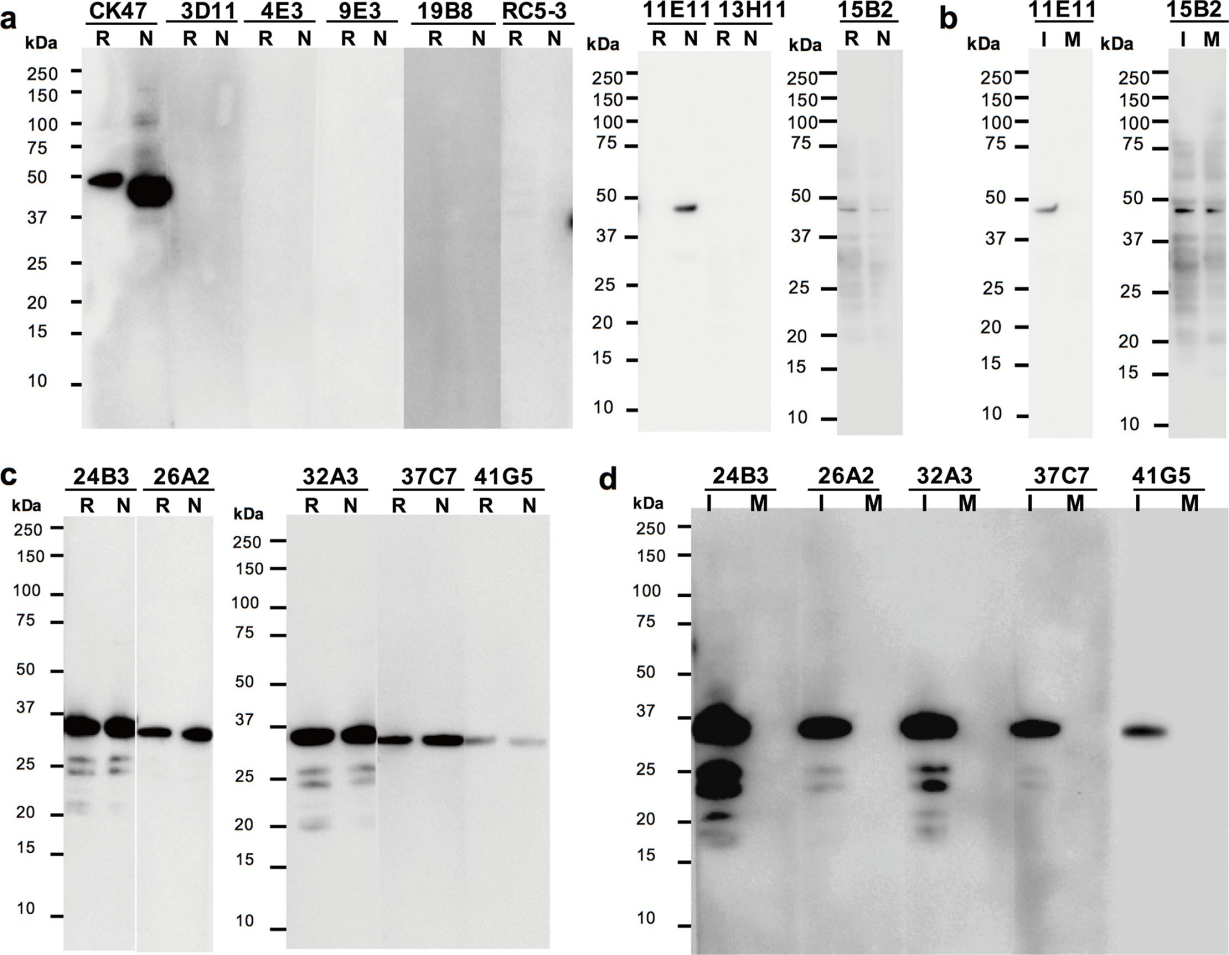
Western blot analysis of mAbs against CHIKV. Western blotting was performed using the indicated anti-CHIKV monoclonal antibodies (mAbs); labelling was detected with horseradish peroxidase (HRP) -conjugated secondary antibody and enhanced chemiluminescence (ECL) substrate. (**a and c**) Western blot profile of mAbs against CHIKV (CP10 strain) -infected Vero cell lysate under reducing (R) and non-reducing (N) conditions from immunization strategy B (panel a) and strategy C (panel c). Anti-CHIKV E1 protein, mAb CK47, was used as an experimental control. (**b and d**) Western blot profile of mAbs against CHIKV (CP10 strain) -infected (I) and mock-infected (M) Vero cell lysate from immunization strategy B under non-reducing conditions (panel B) and from strategy C under reducing conditions (panel d). After the transfer, the PVDF membranes were sliced for individual mAb reactions, with each slice spanning two lanes. Images of slices represent two independent experiments.

To determine the linear epitopes targeted by the new mAbs, western blot analysis was conducted under reducing (R) and non-reducing (N) conditions. Specifically, proteins of CHIKV (CP10 strain) -infected Vero cell lysates boiled in the presence and absence of β-mercaptoethanol (R and N, respectively) were separated by electrophoresis and transferred to PVDF membranes; the resulting blots were probed with individual mAb-containing culture supernatants. One mAb derived from strategy B (clone 11E11) reacted with an approximately 48-kDa protein, corresponding to CHIKV E1, under non-reducing conditions (Fig 3A). All six clones (24B3, 26A2, 32A3, 35B9, 37C7, and 41G5) derived from immunization strategy C reacted with an approximately 35-kDa protein, corresponding to the CHIKV capsid protein, under both reducing and non-reducing conditions (Fig 3C). For all seven of these clones, the reactions reflected specificity for CHIKV proteins, given that these clones did not react with a mock-infected (M) cell lysate (Fig 3B and 3D). With the exception of 41G5, clones obtained via immunization strategy C showed low reactivity against bands below 35 kDa in the CHIKV-infected cell lysate (Fig 3C and 3D). On the other hand, 5 clones obtained via strategy B (3D11, 4E3, 9E3, 19B8, RC3-5, and 13H11) reacted with neither CHIKV-infected nor mock-infected cell lysates. However, one clone derived from strategy B (15B2) reacted with several proteins from both CHIKV-infected and mock-infected cell lysates (Fig 3A and 3B).

### Regions of CHIKV proteins targeted by mAbs

**Fig 4:**
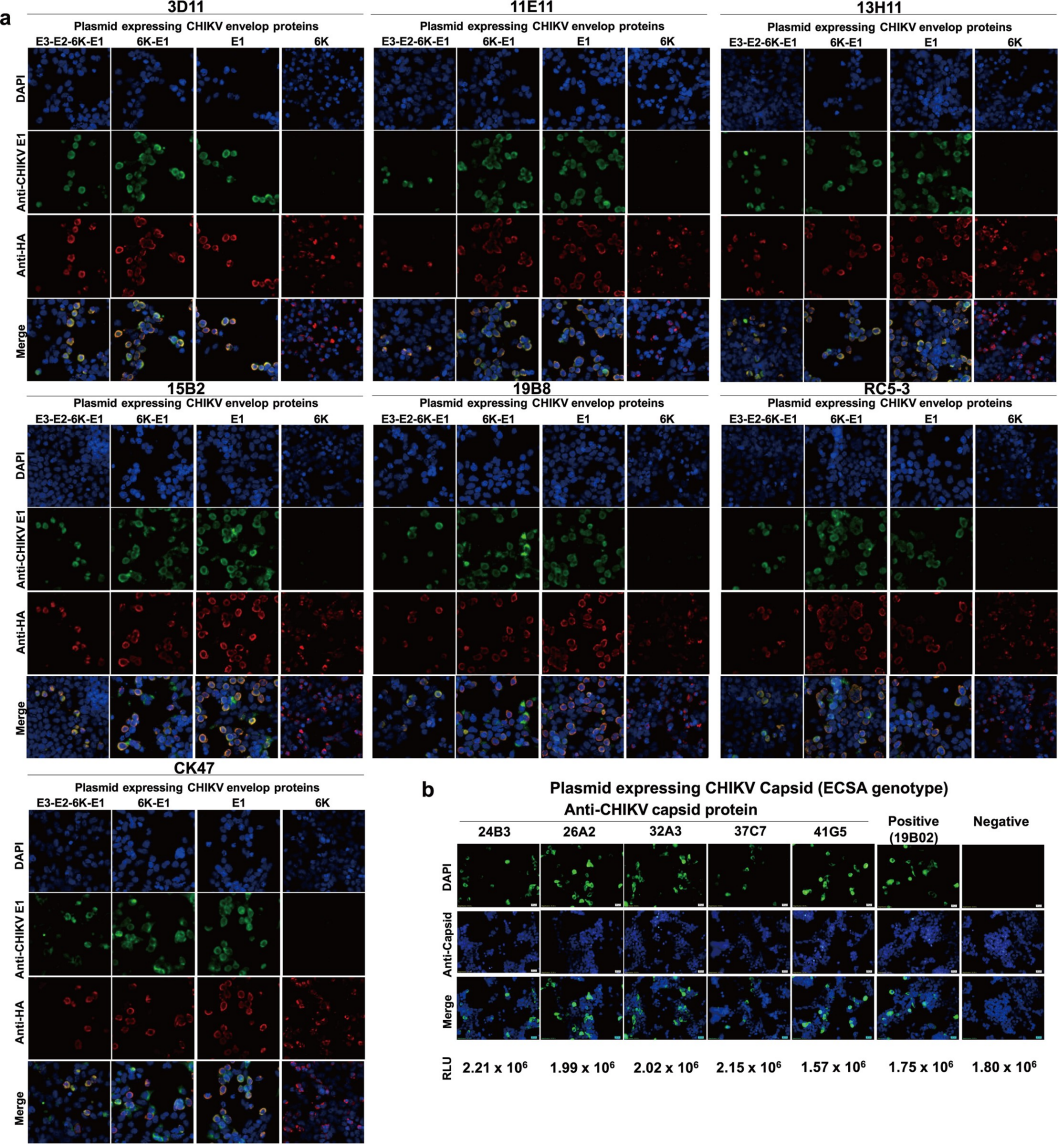
Protein regions targeted by monoclonal antibodies (mAbs) raised against chikungunya virus (CHIKV). **(a)** Indirect immunofluorescent test of mAbs from strategy B against cells transfected with plasmids encoding HA-tagged full-length or truncated versions of CHIKV (CP10 strain) E3-E2-6K-E1, 6K-E1, E1, or 6K envelope proteins. CK47 was used as anti-E1 control antibody. **(b)** mAbs from strategy C against cells transfected with plasmid encoding capsid protein of CHIKV (CP10 strain). 19B02 was used as an anti-C control antibody. Cells that reacted with the 2^nd^ antibody only were used as a negative control. Luciferase counts within culture supernatants expressed from a co-transfected luciferase-secreting plasmid (pMetLuc-Control, Ready-To-Glow Secreted Luciferase Reporter Assay, Takara Bio USA, Inc.) are shown. The reactivity of each mAb was detected by Alexa Fluor 488-conjugated secondary antibody (green color); expression of the HA peptide was detected by Alexa Fluor 555-conjugated secondary antibody (orange/red color); DAPI DNA counterstain was used to stain nuclei of cells (blue color). Images are representative of results obtained from two independent experiments and were taken under 40x objective magnification using an Axio observer Z.1 microscope (Carl Zeiss). The multiple-color images were generated by merging using ImageJ 1.5Oi (National Institutes of Health USA).

As shown in Fig 3, the mAbs generated in the present study recognized proteins of different sizes. To confirm their binding specificity, we first tested (by IIFT) reactivity of the mAbs from strategy B with cells transfected with plasmids encoding HA-tagged full-length (E3-E2-6K-E1) or truncated (6K-E1, 6K, or E1) versions of the CHIKV (CP10-strain) envelope protein. We excluded clones 4E3 and 9E3 from this assay, given those mAbs’ relatively low reactivity in IIFT and lack of specific binding in western blot analysis. As shown in Fig 4A, in accordance with the control anti-CHIKV E1 (clone CK47), our IIFT results demonstrated that mAbs 3D11, 11E11, 13H11, 15B2, 19B8, and RC5-3 were able to recognize cells transfected with plasmids encoding CHIKV envelope proteins, including E3-E2-6K-E1, 6K-E1, and E1. Notably, none of the anti-CHIKV mAbs tested here recognized the (HA-tagged) 6K protein, the expression of which was confirmed by staining with an anti-HA antibody.

We next used IIFT to confirm the targets of the mAbs derived from strategy C. Purified mAbs of five such clones (24B3, 26A2, 32A3, 37C7, and 41G5) were tested for reactivity against cells transfected with a plasmid encoding the CHIKV C protein (CP10 strain). As expected, each of these 5 clones recognized the CHIKV C protein (Fig 4B), as did a commercially available anti-CHIKV C protein antibody, 19B02. Luciferase counts within culture supernatants expressed from a co-transfected luciferase-secreting plasmid (pMetLuc-Control, Ready-To-Glow Secreted Luciferase Reporter Assay, Takara Bio USA, Inc.) verified that transfection efficiency was virtually the same among different wells in the 96-well plates (Fig 4B).

Taken together, these data demonstrated the successful production of two panels of mAbs with reactivity against structural proteins of CHIKV. Specifically, mAbs derived from immunization strategy B targeted the E1 protein, while those derived from strategy C targeted the C protein. Results from western blot analysis of CHIKV-infected cells and IIFT of plasmid-transfected cells also are summarized in Table 2.

### Anti-CHIKV E1 mAbs are unable to inhibit virus entry during infection

**Fig 5:**
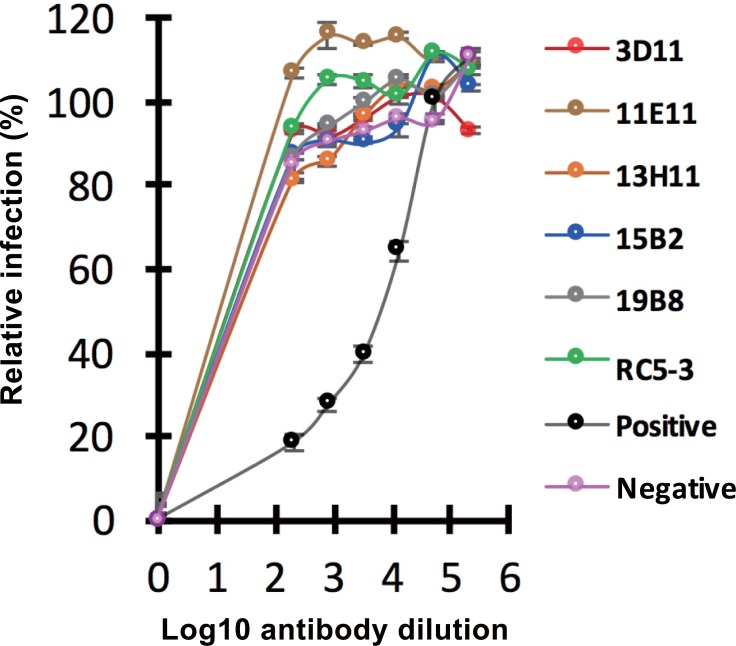
Neutralization profile of anti-CHIKV envelope monoclonal antibodies (mAbs). Six anti-CHIKV E1 mAbs [clones 3D11 (red), 11E11 (brown), 13H11 (orange), 15B2 (blue), 19B8 (gray), and RC5-3 (green)] were tested for their neutralization activity. CK47, which was previously shown to exhibit neutralizing activity in this system, was used as a positive control (black), while 24B3, an anti-capsid protein antibody, was used as a negative control (pink). Four-fold serial dilutions (ranging from 1:200 to 1:51200) of each mAb were incubated with Asian-genotype CHIKV-pseudotyped lentiviral particles prior to infection of U251 cells. Luciferase activity (expressed as relative luciferase units, RLUs), reflecting infectivity of pseudotyped lentiviruses, was measured at 72 hours post-infection. The graph displays the mean relative infectivity of pseudotyped lentiviruses following pre-incubation with various dilutions of mAbs. Error bars indicate standard error of the mean (SEM) from three independent experiments, each performed in duplicate.

Six anti-CHIKV E1 mAb clones were tested for their ability to neutralize viral infection at the entry step. For this purpose, we employed a system of pseudotyped lentiviral particles harboring a *luciferase* reporter and envelope proteins of Asian-genotype CHIKV (strain CK12-686). Four-fold serial dilutions of mAbs, at concentrations ranging from 1:200 to 1:204800, were prepared and individually incubated with pseudotyped lentiviral particles prior to infection of U251 cells. Anti-CHIKV E1 antibody (clone CK47), previously shown to exhibit neutralizing activity in this particular assay [33], was used as a positive control, while anti-CHIKV C protein antibody (clone 24B3) was used as a negative control. Viral infection following pre-incubation with mAbs was normalized against infection following pre-incubation without mAbs (Fig 5). The results indicated that none of the six mAbs that targeted the CHIKV envelope protein blocked entry of CHIKV-pseudotyped lentiviral particles.

### Broad reactivity of anti-E1 protein mAbs towards three genotypes of CHIKV

To determine whether our newly generated mAbs against CHIKV E1 protein (3D11, 11E11, 13H11, 15B2, 19B8, and RC5-3) showed broad-spectrum reactivity towards all three CHIKV genotypes, we first tested their reactivity (using IIFT) against Vero cells infected by each of three strains of CHIKV: CHIKV CP10 (ECSA-IOL), Ross Low psg (ECSA), and ARUBA-1125 (Asian) (Table 3 and S2 Fig). Similar to the results shown in Fig 2, the reactivity of individual mAbs towards CHIKV-infected cells varied among clones. However, each of the newly produced anti-CHIKV E1 mAbs, as well as CK119, showed comparable reactivity towards the CP10, Ross Low psg, and ARUBA-1125 strains (Table 3 and S2 Fig). In contrast, CK47 strongly reacted with CP10 but only weakly reacted with Ross Low psg and ARUBA-1125, as we reported previously [33]. Reactivity against Asian CHIKV ARUBA-1125 was further confirmed by Enzyme-Linked Immuno-Sorbent Assay (ELISA) using concentrated ARUBA-1125 virions as immobilized antigens (S3 Fig), although CK47 showed reactivity against ARUBA-1125 comparable to that obtained with the newly generated mAbs. It is possible that epitopes recognized by the newly generated mAbs are masked by immobilization and/or blocking procedures of the ELISA protocol, although the epitope recognized by CK47 was not affected by those procedures (given that CK47 previously was shown to react strongly with immobilized CHIKV in ELISA) [27].

**Table 3:** Reactivity profile of anti-CHIKV mAbs against chikungunya virus-infected cells.

Immunization strategy	mAb	Reactivity test		
		Chikungunya virus strain		
		CP10	Ross Low psg	Aruba- 1125
B	3D11	++	++	++
	11E11	++	++	++
	13H11	++	++	++
	15B2	++++	++++	++++
	19B8	+++	++	+++
	RC5-3	+	++	++
C	24B3	+++	+++	+++
	26A2	++++	++++	++++
	32A3	++++	+++	+++
	37C7	+++	+++	++
	41G5	++++	+++	+++
Control mAb	CK47^a^	+++	+	+
Control mAb	CK119^b^	++	+++	+++

*Note*; Alexa Fluor 488 signals of each photo were quantified by NIS-Elements software (Nikon Corporation, Tokyo, Japan). ++++ (very strong; mean fluorescence intensity/pixel ≥ 60), +++ (strong; ≥ 35), + +(medium; ≥ 20), + (weak; ≥ 15)

^a^ Control mAb showing the restricted reactivity toward CHIKV ECSA genotype bearing glutamic acid at position 350 of 6K-E1 protein (E350)

^b^ Control mAb against CHIKV E1 protein.

**Fig 6:**
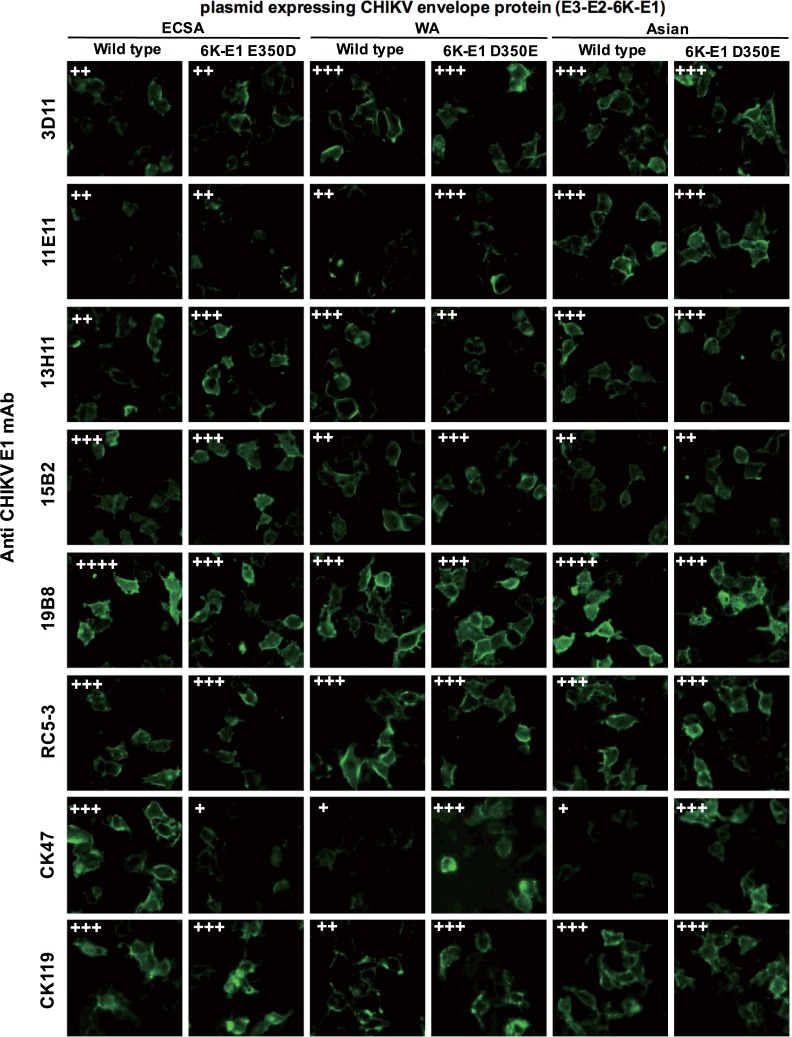
Broad reactivity of anti-E1 protein monoclonal antibodies (mAbs) towards three genotypes of CHIKV. Plasmids were constructed to encode the full-length CHIKV envelope protein (E3-E2-6K-E1) from each of the three viral genotypes: ECSA (CP10 strain), WA (37997 strain), or Asian (CK12-686 strain; Wild type). Site-directed mutagenesis was used to introduce mutations at position 350 of the 6K-E1 proteins in all three genotypes, resulting in ECSA and WA bearing E350D (6K-E1 E350D) and Asian bearing D350E (6K-E1 D350E). Indirect immunofluorescence test was performed by using the indicated anti-E1 mAbs and Alexa Fluor 488-conjugated secondary antibody towards HEK293T cells transfected with each of these six versions of plasmids encoding CHIKV envelope proteins. CK47 was used as a reference for 350E-restricted reactivity, while CK119 was used as the transfection control. Alexa Fluor 488 signals in each photo were quantified by NIS-Elements software (Nikon Corporation, Tokyo, Japan). ++++ (very strong; mean fluorescence intensity/pixel ≥ 75), +++ (strong; ≥ 35), ++ (medium; ≥ 26), + (weak; ≥ 15). Images shown are representative of results obtained from two independent experiments and were taken under 40x objective magnification using an IX71 microscope (Olympus, Tokyo, Japan).

We then examined reactivity (using IIFT) against cells transfected with plasmids expressing full-length CHIKV envelope proteins (E3-E2-6K-E1) from each CHIKV genotype, including those from ECSA (strain CP10), WA (strain 37997), and Asian (strain CK12-686) genotypes. We found that all six clones of the newly generated anti-E1 mAbs exhibited comparable binding activity towards each of the three genotypes, although there were small differences in reactivities among the clones (Fig 6). As reported previously, clone CK47 (included as a positive control) showed strong reactivity against ECSA E1 but only weak reactivity against the Asian and WA E1s [33].

We previously showed that CK47 reacted strongly with 6K-E1 protein harboring a glutamic acid (E) at amino acid position 350 but only reacted weakly with 6K-E1 protein harboring an aspartic acid (D) at this position [33]. We further explored the binding reactivity of our newly produced mAbs towards 6K-E1 proteins selectively mutated at amino acid 350. Specifically, plasmids encoding mutant CHIKV envelope proteins (E3-E2-6K-E1) of each of the three genotypes (E350D-mutated ECSA-genotype 6K-E1 protein, and D350E-mutated WA- and Asian-genotypes 6K-E1 proteins [33]) were transfected into 293T cells, and mAb reactivity then was examined by IIFT together with the semi-quantitative measurement of the signal detected in individual photos (Fig 6). CK47 was used as a reference for 350E-restricted reactivity, while CK119 was used as the transfection control. As expected, CK47 demonstrated low reactivity against cells transfected with the plasmids encoding 6K-E1 with D at position 350 (including the ECSA mutant (6K-E1 E350D) and the unmutated WA and Asian 6K-E1 proteins) compared to reactivity against cells transfected with the plasmids encoding 6K-E1 with E at position 350 (including the unmutated ECSA protein and the WA and Asian mutants (6K-E1 D350E)). As shown in Fig 6, reactivity against 6K-E1 proteins of the new mAbs were not affected by mutation at amino acid 350.

### Broad reactivity of anti-capsid mAbs towards CHIKV genotypes

**Fig 7:**
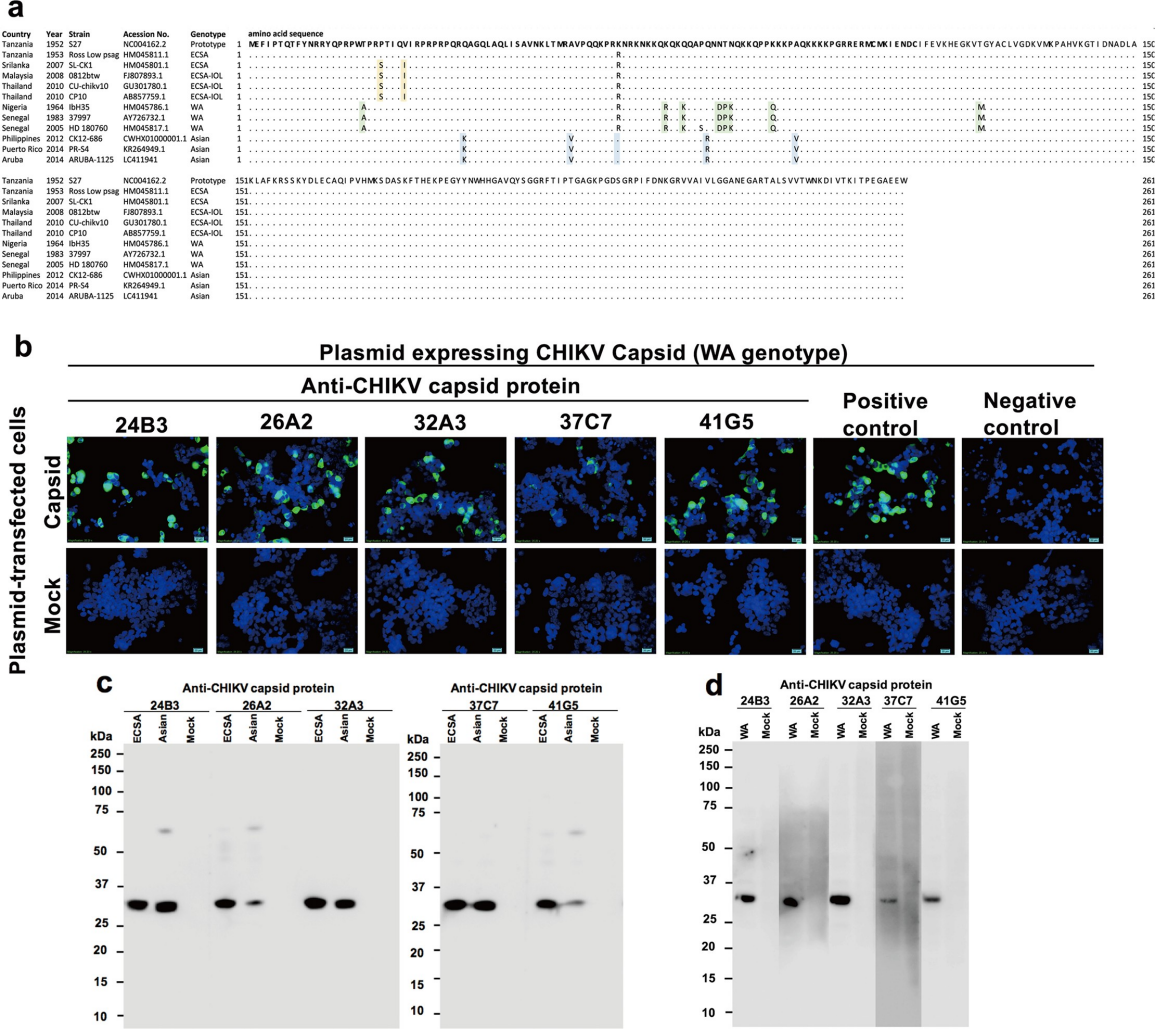
Broad reactivity of anti-CHIKV capsid monoclonal antibodies (mAbs) against the amino acid variation among genotypes. **(a)** Amino acid variation of CHIKV capsid protein. To analyze amino acid variation among genotypes, multiple sequence alignment was performed using the Molecular Evolutionary Genetic Analysis (MEGA) software, version 7.0. The first 131 amino acid residues shown in bold indicate the N-terminal portion, while the remaining residues (aa 132–261) correspond to the C-terminal portion of the CHIKV capsid protein. The amino acids highlighted in yellow, green, and blue indicate the ECSA-, WA-, and Asian-genotype-specific amino acid residues. **(b)** Reactivity test of anti-CHIKV capsid protein mAbs towards the WA genotype by indirect immunofluorescence test. Five mAbs were tested against HEK293T cells transfected with plasmids encoding the CHIKV capsid protein of the WA genotype. 19B02 was used as an anti-C control antibody. Cells that reacted with the 2^nd^ antibody only were used as a negative control. The reactivity of each mAb was detected by the green labeling indicating Alexa Fluor 488-conjugated secondary antibody. Images were taken under 40x objective magnification using an Axio observer Z.1 microscope (Carl Zeiss). **(c)** Linear epitope-binding profiles of anti-CHIKV capsid protein antibodies by western blot analysis. The five indicated mAbs were tested against concentrated chikungunya virions of ECSA (CP10 strain) and Asian (ARUBA-1125) -genotype viruses under reducing conditions. Concentrated culture supernatants of mock-infected cells served as a negative control (Mock). **(d)** The five indicated mAbs were tested against the lysate of HEK293T cells transfected with a plasmid encoding the CHIKV capsid protein of the WA genotype (37997 stain) or mock-transfected cells. Antibody labeling was detected with horseradish peroxidase (HRP) -conjugated secondary antibody and was visualized with enhanced chemiluminescence (ECL) substrate. Transferred PVDF membranes were sliced for individual mAb reaction and re-combined during the detection process.

To know whether the newly generated anti-capsid mAbs also show broad reactivity against all three CHIKV genotypes, we first examined reported variations among the C proteins of different CHIKV strains. The CHIKV C protein comprises 261 amino acid residues. The N-terminal 113 residues are involved in viral RNA binding as well as nucleocapsid oligomerization. The C-terminal portion, extending from residues 114 to 261, forms capsomeres and is present in the outer shell, covering the nucleocapsid core [29]. To explore amino acid variation of the C protein among CHIKV of different genotypes, we retrieved (from the NCBI database) the amino acid sequence of this protein in CHIKV strains selected to represent each genotype, and subjected these protein sequences to multiple sequence alignment. We also determined the nucleotide sequence of the C-encoding gene of the ARUBA-1125 strain of CHIKV and incorporated the corresponding deduced amino acid sequence into the multiple alignment. As shown in Fig 7A, using the C protein from the prototypical CHIKV S27 strain as the reference sequence, we observed amino acid variation among the 3 genotypes at 6.13% (16 out of 261) of the residues. Of those positions with variation, two (residues 23 and 27) occurred in ECSA and ECSA-IOL strains, five (residues 37, 55, 63, 78, and 93) occurred in Asian strains, and nine (20, 71, 74, 77, 80–82, 99 and 124) occurred in WA strains. Interestingly, all but one of these amino acid variations (15 out of 113 positions, 13.3% variation rate) were located within the N-terminal 113 residues, which function primarily in RNA binding. In contrast, only a single amino acid variation (at position 124; 1 out of 148 positions, or 0.7% variation rate) mapped to the C-terminus, which contributes to the outer shell of the virion; this variation was present only in the WA genotype.

We then tested (using IIFT) reactivity of the anti-C mAbs towards the different genotypes of CHIKV. Notably, two of these five mAbs (clones 24B3 and 26A2) displayed broad reactivity towards Vero cells infected with CHIKV CP10 (ECSA-IOL), Ross Low psg (ECSA), or ARUBA-1125 (Asian) strains. The other three mAbs (clones 32A3, 37C7, and 41G5) showed slightly stronger reactivity against the ECSA genotype, especially the CP10 strain, than against the ARUBA-1125 strains (Table 3 and S4 Fig). Among the three genotypes, the WA genotype showed the highest level of amino acid variation in the C protein compared with the prototype. To determine whether our anti-C mAbs also react with the WA capsid, we constructed a plasmid expressing the WA-genotype (37997 strain) CHIKV C protein and tested reactivity of anti-C mAbs against the transfected cells. We found that all of the anti-C mAb clones were able to recognize the WA-genotype C protein, despite the numbers of amino acid variations in the WA C protein (Fig 7B).

Finally, these mAbs were used as probes in western blot analysis of reactivity towards all three genotypes of CHIKV. For ECSA and Asian genotypes, viral particles of the CP10 and ARUBA-1125 strains, respectively, were normalized to 1x10^5^ PFU/mL and subjected to western blot analysis under reducing conditions. Since a live WA-genotype virus was not available in the present study, we used a lysate of cells transfected with a plasmid encoding the capsid protein of CHIKV strain 37997 for this analysis. Our western blot results showed that these mAbs strongly recognized C protein (a band running at 35 kD), both in samples of live virus (ECSA and Asian genotypes; Fig 7C) and the lysate of cells expressing the WA C protein (Fig 7D). Taken together, these results demonstrated that all five anti-C mAbs exhibited broad reactivity towards all three genotypes of CHIKV. Furthermore, two of the five mAbs showed no apparent difference in reactivity against the C proteins from CHIKVs of different genotypes.

### Cross-reactivity of anti-CHIKV mAbs towards other viruses

We evaluated (using IIFT) the cross-reactivity of the newly isolated anti-CHIKV mAbs against the closely related alphaviruses ONNV, MAYV, RRV, VEEV, EEEV, SINV, and WEEV (Table 4, S5 Fig, S6 Fig, S7 Fig, S8 Fig, S9 Fig, S10 Fig, S11 Fig, S12 Fig and S13 Fig). We found that two of the anti-E1 antibodies (3D11 and 15B2,) lacked cross reactivity with any of the alphaviruses we examined (Table 4, S6 Fig, S7 Fig, S8 Fig, S9 Fig, S10 Fig, S11 Fig, S12 Fig, S13 Fig). Therefore, we concluded that these two antibodies were CHIKV-specific. In addition, another two anti-E1 antibodies (19B8, and RC5-3) showed weak cross reactivity only with ONNV (Table 4 and S6 Fig). In contrast, anti-E1 antibodies 11E11 and CK119 cross-reacted with all of the alphaviruses examined (Table 4, S6 Fig, S7 Fig, S8 Fig, S9 Fig, S10 Fig, S11 Fig, S12 Fig and S13 Fig). One of the anti-E1 antibodies (13H11) and all of the anti-C antibodies (24B3, 26A2, 32A3, 37C7, and 41G5) cross-reacted only with ONNV (Table 4 and S6 Fig). This result is consistent with the fact that the C proteins of CHIKV and ONNV share more than 88% identity at the amino acid level, and that E1 proteins in these 2 viruses share more than 87% amino acid identity. However, it is a surprising that CK47 cross-reacted with ONNV, RRV, and two strains of VEEV (Table 4, S6 Fig, S8 Fig, S9 Fig and S10 Fig), since all of these alphaviruses carry an aspartic acid at the aa residue 350 of E1.

**Table 4:** Cross-reactivity profiles of anti-CHIKV mAbs against cells infected with other alphaviruses.

Immunization strategy	mAb	alphaviruses									
		CHIKV	ONNV	MAYV	RRV	VEEV 78v	VEEV TrD	EEEV	SINV	WEEV	Mock
B	3D11	+	-	-	-	-	-	-	-	-	-
	11E11	++++	+++	+	++	++	+++	++++	++	++	-
	13H11	++	+	-	-	-	-	-	-	-	-
	15B2	+++	-	-	-	-	-	-	-	-	-
	19B8	++	+	-	-	-	-	-	-	-	-
	RC5-3	+	+	-	-	-	-	-	-	-	-
C	24B3	++++	+++	-	-	-	-	-	-	-	-
	26A2	++++	+++	-	+	-	-	-	-	-	-
	32A3	+++	+++	-	-	-	-	-	-	-	-
	37C7	+++	+++	-	-	-	-	-	-	-	-
	41G5	++++	++	-	-	-	-	-	-	-	-
Control mAb	CK47^a^	++++	+++	-	+	+++	+++	-	-	-	-
Control mAb	CK119^b^	+++	+++	++	++	++	+++	++++	+++	++	-
Negative control ^c^		-	-	-	-	-	-	-	-	-	-

*Note*; Alexa Fluor 488 signals of each photo were quantified by NIS-Elements software (Nikon Corporation, Tokyo, Japan). ++++ (very strong; mean fluorescence intensity/pixel ≥ 75), +++ (strong; ≥ 50), + +(medium; ≥ 35), + (weak; ≥ 25),—(negative; <25)

^a, b^ Control mAbs for CHIKV E1

^c^ Second antibody only; Alexa Fluor 488-conjugated goat-anti mouse IgG.

We then evaluated the cross-reactivity of the newly isolated anti-CHIKV mAbs against flaviviruses [including DENV serotype 1 (Mochizuki), 2 (16681), 3 (H87), and 4 (H241), as well as ZIKV (SV0010/15)] that have overlapping geographical and clinical presentations with CHIKV. mAb 4G2 was used as a positive control for detection of DENV and ZIKV. Notably, despite their broad reactivity towards all 3 CHIKV genotypes, none of our mAbs displayed apparent cross-reactivity against DENV or ZIKV (Table 5, S16 Fig and S17 Fig).

**Table 5:** Cross-reactivity profile of anti-CHIKV mAbs against flavivirus-infected cells.

Immunization strategy	mAb	Cross reactivity test	
		DENV 1–4	ZIKV (SV0010/15)
B	3D11	-	-
	11E11	-	-
	13H11	-	-
	15B2	-	-
	19B8	-	-
	RC5-3	-	-
C	24B3	-	-
	26A2	-	-
	32A3^a^	-	-
	37C7	-	-
	41G5	-	-
Control mAb	4G2a	+	+

Alexa Fluor 488 signals of each photo were quantified by NIS-Elements software (Nikon Corporation, Tokyo, Japan). Note; + (positive; mean fluorescence intensity/pixel ≥ 15),—(negative; < 5)

^a^ Positive control for *Flaviviruses* (DENV and ZIKV)

## Discussion

In the present study, we generated mouse anti-CHIKV mAbs that have broad reactivity towards all three genotypes of CHIKV; such mAbs might be of great value for further diagnostic test development. Using three strategies of immunization in combination with hybridoma technology, we generated new panels of mouse mAbs against the CHIKV E1 and capsid structural proteins. These mAbs recognized all three genotypes of CHIKV currently responsible for chikungunya outbreaks worldwide. We also verified that four of our new mAbs lacked cross reactivity with or significantly less affinity for the most closely related alphavirus, ONNV.

Although studies have been conducted on both human and mouse mAbs against CHIKV, most of that work focused on protective and therapeutic applications [43–46]. One paper described the production and characterization of anti-CHIKV antibodies for diagnostic applications, but that study focused on the development of an anti-CHIKV E2 protein for use in an ELISA platform [47]. Masrinoul *et al*. [27] and Okabayashi *et al*. [28] produced and applied mouse anti-CHIKV E1 for diagnostic application in the rapid IC platform, a simple and inexpensive technology. Although the IC device showed excellent performance against ECSA-genotype CHIKV, further improvement was needed for application in detecting CHIKV of the other two genotypes (WA and Asian); our previous study revealed that the low sensitivity of this IC test against WA and Asian stemmed from one of mAbs in the IC platform [33]. As we described in the present work, we conducted research focusing on mAb production and characterization to overcome the obstacle found in the previous IC test.

Among the CHIKV structural proteins, E2 and E1 are envelope proteins present on mature virions [14, 15]. E2 is known to act as a receptor-binding protein and is exposed on the periphery of the CHIKV particle [48]. E2 has high immunogenicity and is a target for current vaccine development [45]. However, as a target for antigen detection, E2 presents several challenges. Notably, among the CHIKV structural proteins, E2 shows the highest degree of amino acid variation. A study conducted by Schuffenecker *et al*. comparing CHIKV isolated during the Indian Ocean outbreak with the prototype ECSA-genotype virus (S27 strain) revealed that the protein divergence of IOL from the prototype ECSA stain was 3.3% in the E2 glycoprotein, while divergence was only 0.68% in the E1 protein [4]. Moreover, these variation rates were even higher when comparing among CHIKV of different genotypes. Our previous sequence comparison among 6 ECSA strains, 5 ECSA-IOL strains, 5 WA strains, and 5 Asian strains of CHIKV revealed variations of 7.7% and 4.3% in E2 and E1, respectively [33]. E2 possesses a larger number of B-cell epitopes targeted by human anti-CHIKV neutralizing antibodies than does E1 [22, 49], thus increasing the possibility of selection for amino acid changes in the targeted epitopes. The greater antigenicity of CHIKV E2 is consistent with a similar result in SINV. Specifically, SINV E1, a class-II fusion protein, is centrally located and is covered by SINV E2 [48]. The dissociation of the SINV E1 from E2 occurs only upon membrane fusion in endosomes, under low pH conditions [50].

Beyond the possibility of amino acid variations selected during immune evasion, increased immune responses, especially Ig-mediated responses, may directly impact the sensitivity of antibody-based immunodiagnostic platforms. As has been confirmed in the detection of the dengue non-structural protein 1 (NS1), the dissociation of NS1 and an anti-NS1 antibody improved the sensitivity of an antigen detection assay [51]. Anti-CHIKV E2 antibodies are likely to be present in the circulation of CHIKV-infected individuals, given that the E2 protein has more B-cell epitopes than does E1. Therefore, in the present study, two immunization strategies, A and B, were employed with the intent of identifying anti-CHIKV E1 antibodies rather than anti-E2 antibodies. This approach was expected to minimize the chance of recognition failure caused by mutation due to viral evolutionary forces, as well as avoiding potential interference by immune complexes.

Among the three immunization strategies employed in the present study, immunization strategy A did not result in any single clone isolates when screening against cells transfected with a plasmid encoding the Asian-genotype CHIKV 6K-E1 protein (Table 1 and S1 Fig). In the field of immunogen design in vaccine development, the whole organism (in theory) provides enhanced immunogenicity and evokes stronger immune reactions than those evoked by partial sub-units of the pathogen [52]. It is thus likely that immunization employing two rounds of boosting with SeV-infected cells increased the immune response against SeV but not that against CHIKV. In immunization strategy B, SeV/Asian-genotype 6K-E1 was administered to a mouse previously dosed twice with whole inactivated CHIKV, resulting in the generation of several clones that recognized the E1 protein (Table 1). As expected, immunization strategy C, which employed three doses of whole inactivated CHIKV, gave rise to the highest number of antigen-specific Ig clones, all of which were later shown to recognize the CHIKV capsid protein (Table 1). However, it remains unclear why strategy C failed to elicit mAbs against CHIKV E1 protein, given that a similar strategy previously yielded clones (CK47 and CK119) with activity against CHIKV E1 [27].

Our western blot results showed that, in contrast to the anti-CHIKV C mAbs, most of the anti-CHIKV E1 mAbs were unable to recognize CHIKV-infected Vero cell lysates separated under either reducing- or non-reducing conditions. This result suggested that the target of these mAbs is a conformational epitope that does not persist in the denatured protein. Although one mAb, 11E11, failed to recognize E1 under reducing conditions, this mAb was able to bind E1 protein under non-reducing conditions. Based on the sequence of the ECSA-genotype CHIKV (CP10 strain) used in the present analysis, we previously demonstrated that the correct folding of CHIKV E1 during maturation in the ER involves 16 cysteine residues that form eight disulfide bridges [53]. It is interesting to note that the E1 proteins of alphaviruses undergo at least three distinct conformational changes, with different numbers of disulfide bonds present in each intermediate [54]. In the presence of β-mercaptoethanol, disulfide bonds will be reduced, resulting in tertiary structure destabilization of proteins containing disulfide bonds, and eventually leading to abolishment of these proteins’ immunological properties [55]. This fact may explain why one of our anti-E1 protein mAbs (clone 11E11) was able to react with the E1 protein only under non-reducing conditions. Unlike the E1 protein, the CHIKV capsid protein, a cytosolic protein, requires the activity of a chymotrypsin-like protease domain to facilitate protein folding; thus, maturation of the C protein depends on a autocatalytic cleavage event. Therefore, maturation of the C protein is independent of ER-acquired disulfide bond formation [56].

A multifunctional C protein consists of three regions: region I (residues 1–80), which functions as a non-specific binding site for viral RNA; region II (residues 81–113), which serves as specific binding site for viral RNA; and region III (residues 114–261), which facilitates binding to the cytoplasmic domain of the E2 protein [57, 58]. Consistent with a previous study by Goh *et al*.,[57] our sequence alignment of C proteins from CHIKVs of various genotypes showed that the C protein is more strongly conserved among genotypes than are envelope proteins. The C protein of the intact CHIKV virion is covered by the envelope protein and therefore is unlikely to be recognized by B-cell receptors. However, putative linear B-cell epitopes have been mapped to amino acid residues on CHIKV C protein regions I and III [59]. Several studies have reported on the possible application and diagnostic potential of anti-capsid mAbs [60, 61]. In the present study, anti-CHIKV C protein antibodies were classified into two groups: those showing comparable reactivity among the three CHIKV genotypes (mAbs 24B3 and 26A2) and those with superior reactivity against ECSA compared to CHIKVs of other genotypes (mAbs 32A3, 37C7, and 41G5). Based on the degree of amino acid variation among the CHIKV C proteins of different strains, we hypothesized that the binding epitopes targeted by mAbs 32A3, 37C7, and 41G5 are located in the N-terminal portion (region I or II) of the C protein, regions that showed a higher degree of variation. On the other hand, the highly conserved C-terminal portion (region III) of the C protein is inferred to be recognized by mAbs 24B3 and 26A2. Consistent with this hypothesis, only 26A2 cross-reacted with RRV, a virus that possesses a C protein that shows approximately 72% amino acid identity with that of CHIKV (Table 4, S5 Fig). Considered together, our results demonstrated the successful generation of a panel of anti-CHIKV C protein mAbs; these clones were able to recognize the different CHIKV genotypes, notably including the ECSA and Asian genotypes that currently are in worldwide circulation. Hence, our anti-CHIKV C mAbs are highly promising tools for use in immunodiagnostic test development.

Our immunization strategies successfully produced panels of mouse mAbs recognizing all 3 CHIKV genotypes currently in circulation around the globe. This breakthrough is expected to facilitate overcoming the challenges reported with the previously developed CHIKV antigen-detection IC test [32], which showed diminished sensitivity caused by a single amino acid substitution in the 6K-E1 protein [33]. Most of the new mAbs described here exhibit no cross-reactivity against other arboviruses (such as DENV and ZIKV) that induce similar clinical manifestations, nor against the closely related alphavirus SINV, which is currently the only other alphavirus (aside from CHIKV) that has been found in Asia [62]. Examination of cross reactivity with other alphaviruses such as ONNV, MAYV, RRV, VEEV, EEEV, and WEEV revealed that two of the novel anti-E1 antibodies (3D11 and 15B2) lacked cross reactivity with any of the alphaviruses we examined. In addition, another two anti-E1 antibodies (19B8 and RC5-3) weakly cross-reacted only with the most closely related ONNV. We also identified two clones (11E11 and CK119) that displayed cross-reactivity against all the alphaviruses we examined; this property may be of use for further applications.

In response to the continuing global spread of CHIKV, a diagnostic tool that can accurately detect CHIKV in a simple and inexpensive way is of high importance. Here, we reported the production and characterization of panels of mouse-derived mAbs against CHIKV structural proteins; members of these panels recognized the currently circulating genotypes of CHIKV, including the ECSA, ECSA-IOL, and Asian genotypes. The incorporation of these antibodies targeting CHIKV structural proteins into the IC device, as well as their use in IC evaluation, are in progress. These tools may represent great improvements for clinical diagnosis and surveillance of CHIKV infection.

## Supporting information

S1 Checklist NC3Rs ARRIVE guidelines checklist 2014. (DOCX)

S1 Fig Flow chart of monoclonal antibody selection. (PDF)

S2 Fig Reactivity profile of anti-CHIKV E1 mAbs against CHIKV-infected Vero cells. (PDF)

S3 Fig Enzyme-Linked Immuno-Sorbent Assay (ELISA). (PDF)

S4 Fig Reactivity profile of anti-CHIKV capsid mAbs against CHIKV-infected Vero cells. (PDF)

S5 Fig Indirect immunofluorescence analysis of anti-chikungunya virus (CHIKV) mAbs against CHIKV-infected Vero cells. (PDF)

S6 Fig Indirect immunofluorescence analysis of anti-CHIKV mAbs against O’nyong-nyong virus (ONNV) -infected Vero cells. (PDF)

S7 Fig Indirect immunofluorescence analysis of anti-CHIKV mAbs against Mayaro virus (MAYV) -infected Vero cells. (PDF)

S8 Fig Indirect immunofluorescence analysis of anti-CHIKV mAbs against Ross river virus (RRV) -infected Vero cells. (PDF)

S9 Fig Indirect immunofluorescence analysis of anti-CHIKV mAbs against Venezuelan Equine Encephalitis virus (VEEV) -infected Vero cells. (PDF)

S10 Fig Indirect immunofluorescence analysis of anti-CHIKV mAbs against Venezuelan Equine Encephalitis virus (VEEV) -infected Vero cells. (PDF)

S11 Fig Indirect immunofluorescence analysis of anti-CHIKV mAbs against Eastern Equine Encephalitis virus (EEEV) -infected Vero cells. (PDF)

S12 Fig Indirect immunofluorescence analysis of anti-CHIKV mAbs against Sindbis virus (SINV) -infected Vero cells. (PDF)

S13 Fig Indirect immunofluorescence analysis of anti-CHIKV mAbs against Western Equine Encephalitis (WEEV) -infected Vero cells. (PDF)

S14 Fig Indirect immunofluorescence analysis of anti-CHIKV mAbs against Mock-infected Vero cells. (PDF)

S15 Fig Indirect immunofluorescence analysis of anti-CHIKV mAbs against Sindbis virus-infected BHK cells. (PDF)

S16 Fig Indirect immunofluorescence analysis of anti-CHIKV mAbs against Dengue virus-infected Vero cells. (PDF)

S17 Fig Indirect immunofluorescence analysis of anti-CHIKV mAbs against Zika virus-infected Vero cells. (PDF)
