# Supplementary material for: Broad-spectrum monoclonal antibodies against chikungunya virus structural proteins: Promising candidates for antibody-based rapid diagnostic test development
Source: PLoS One. 2018 Dec 17;13(12):e0208851. doi: 10.1371/journal.pone.0208851 (PMC6296674; doi:10.1371/journal.pone.0208851)

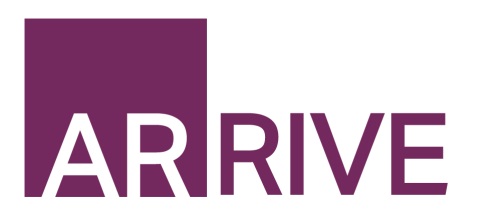


The ARRIVE Guidelines Checklist

Animal Research: Reporting In Vivo Experiments

Carol Kilkenny^1^, William J Browne^2^, Innes C Cuthill^3^, Michael Emerson^4^ and Douglas G Altman^5^

*^1^The National Centre for the Replacement, Refinement and Reduction of Animals in Research, London, UK, ^2^School of Veterinary Science, University of Bristol, Bristol, UK, ^3^School of Biological Sciences, University of Bristol, Bristol, UK, ^4^National Heart and Lung Institute, Imperial College London, UK, ^5^Centre for Statistics in Medicine, University of Oxford, Oxford, UK.*

|  | | ITEM | RECOMMENDATION | Section/ Paragraph |
| --- | --- | --- | --- | --- |
| 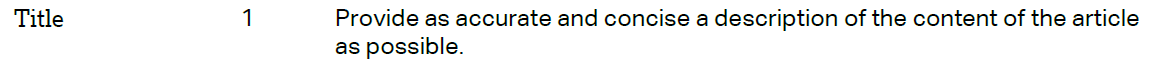 | | | Title |  |
| 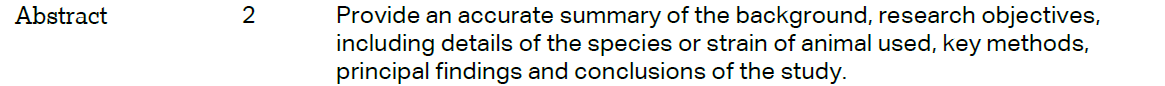 | | | Abstract |  |
| INTRODUCTION | | |  |  |
| 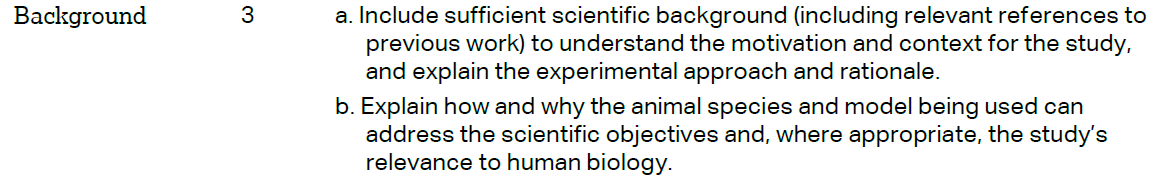 | | | Paragraphs4-7 |  |
| 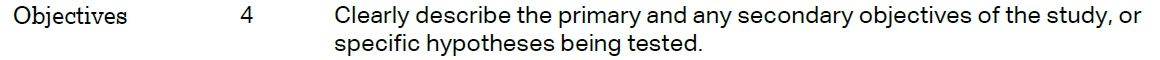 | | | Paragraph 7 |  |
| METHODS | | |  |  |
| 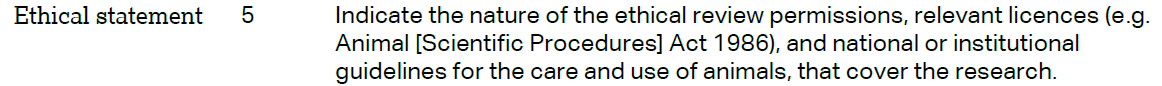 | | | Paragraph 1 “Ethics statement” |  |
| 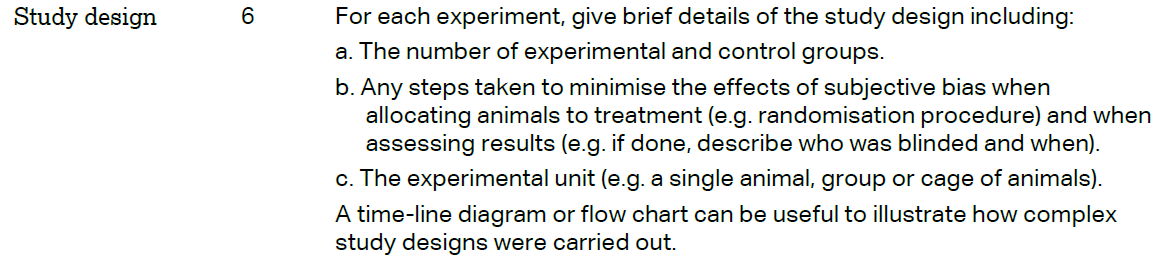 | | | Paragraph 9 “Immunization Strategies and mAb production” |  |
| 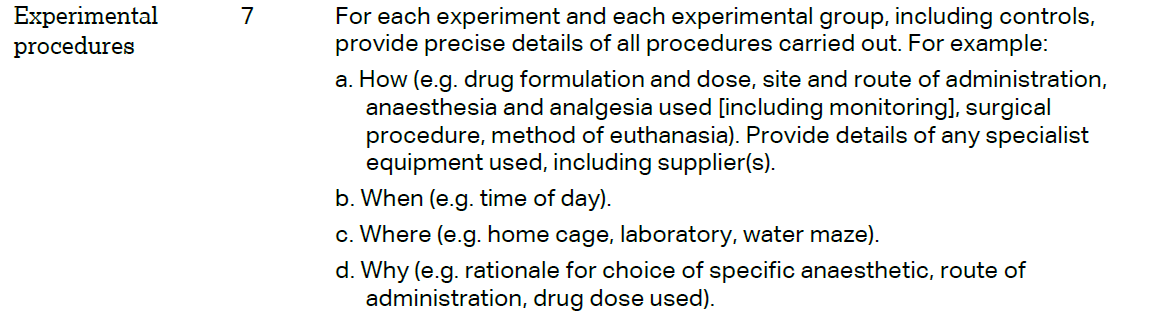 | | | Paragraph 9 “Immunization Strategies and mAb production” |  |
| 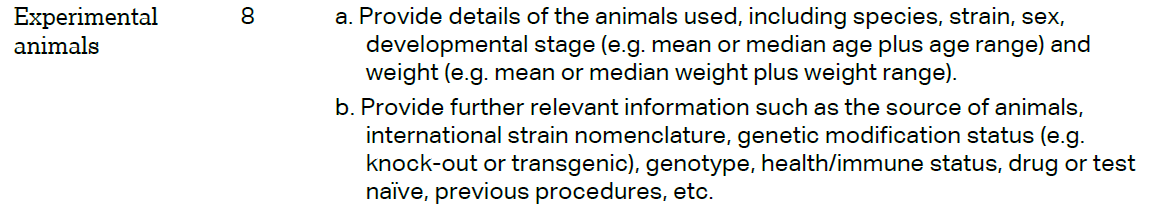 | | | Paragraph 9 “Immunization Strategies and mAb production” |  |

The ARRIVE guidelines. Originally published in *PLoS Biology*, June 2010^1^

| 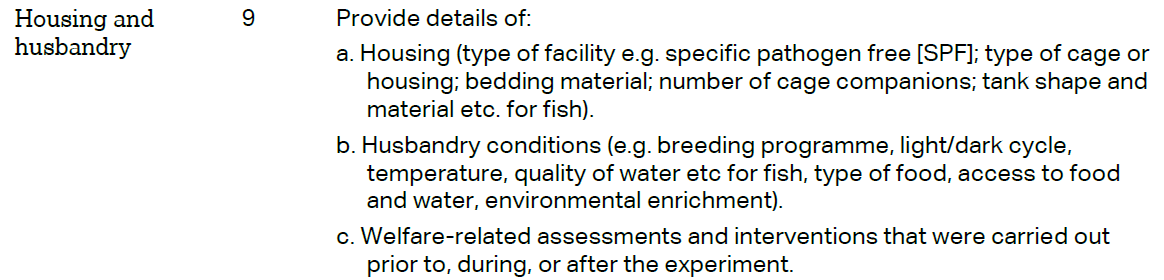 | Paragraph 9 “Immunization Strategies and mAb production” | |
| --- | --- | --- |
| 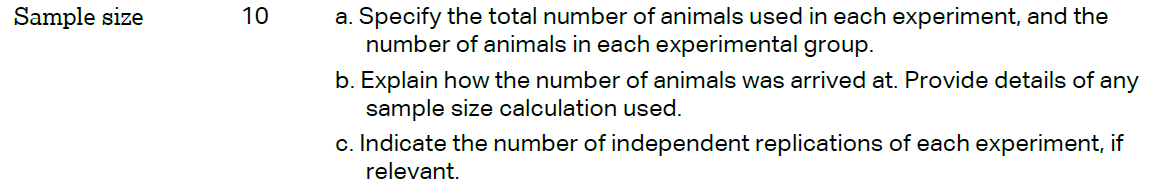 | Paragraph 9 “Immunization Strategies and mAb production” | |
| 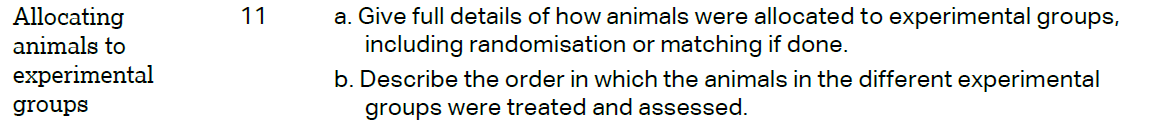 | Paragraph 9 “Immunization Strategies and mAb production”  Table 1 | |
| 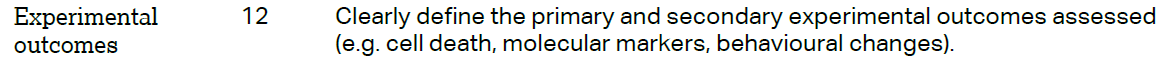 | Paragraph 9 “Immunization Strategies and mAb production” | |
| 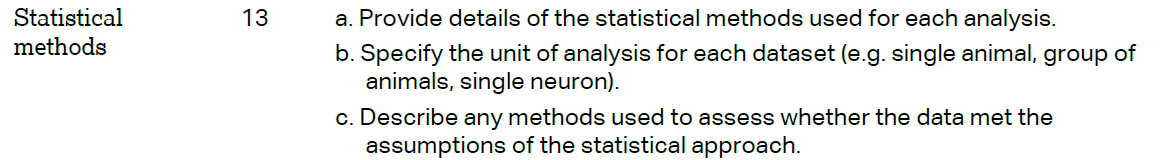 | NA | |
| RESULTS |  | |
| 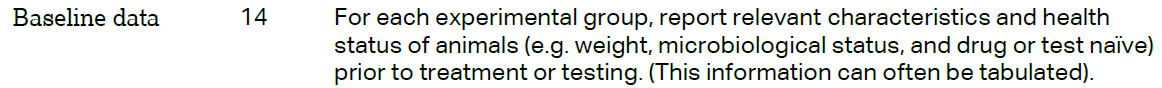 | Paragraph 2  Methods paragraph 9 | |
| 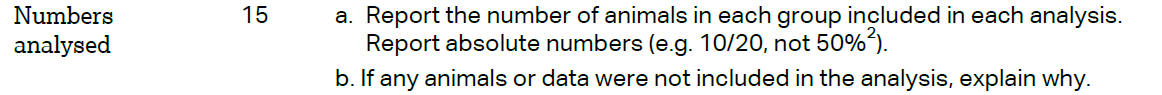 | Paragraph 2 | |
| 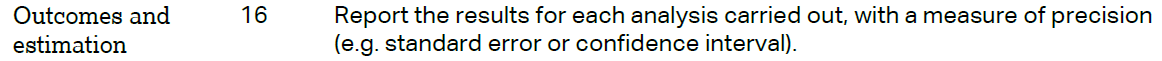 | NA | |
| 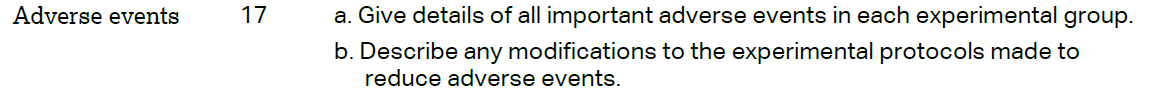 | Methods paragraph 9 | |
| DISCUSSION |  | |
| 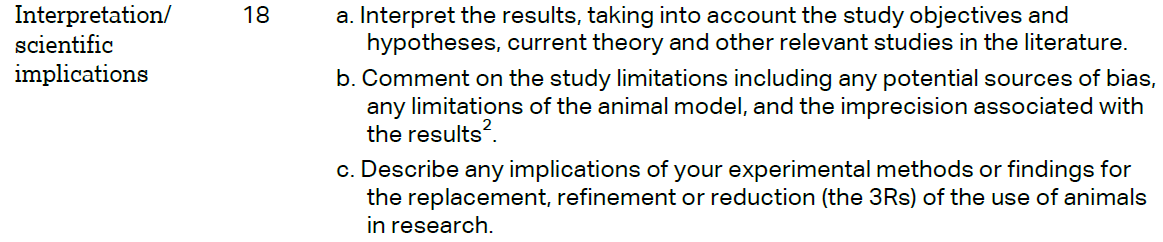 | Paragraphs 4, 5 and 8 | |
| 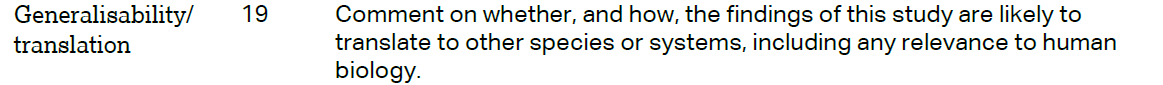 | NA | |
| 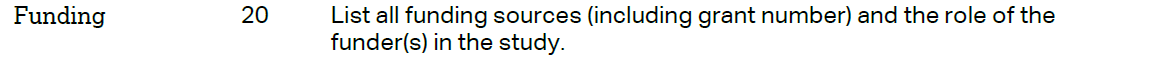 | | “Funding Statement” |


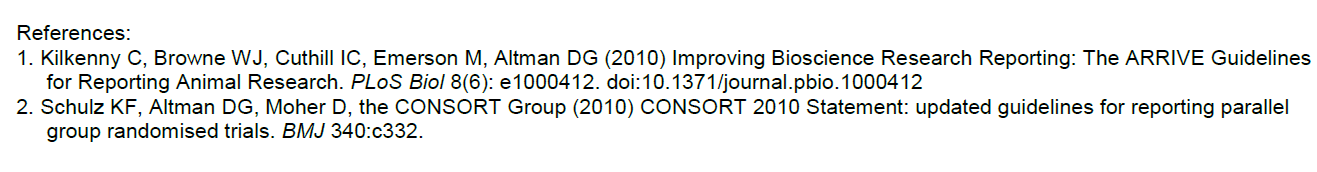

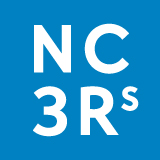

Supplement: S1 Checklist — (DOCX) [file pone.0208851.s001.docx]
