# Supplementary figures and images for: Broad-spectrum monoclonal antibodies against chikungunya virus structural proteins: Promising candidates for antibody-based rapid diagnostic test development
Source: PLoS One. 2018 Dec 17;13(12):e0208851. doi: 10.1371/journal.pone.0208851 (PMC6296674; doi:10.1371/journal.pone.0208851)

**S1 Fig**

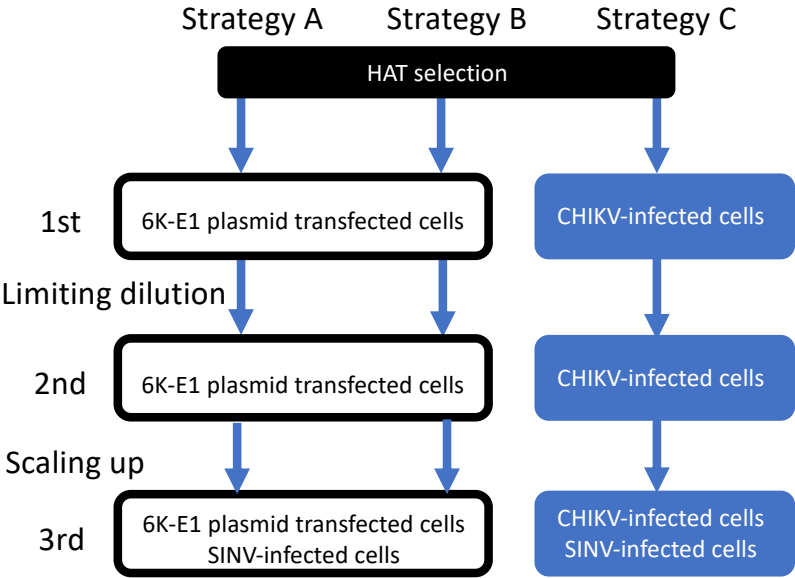

**S1 Fig. Flow chart of monoclonal antibody selection.**

Supplement: S1 Fig — (PDF) [file pone.0208851.s002.pdf]
