# Supplementary material for: Broad-spectrum monoclonal antibodies against chikungunya virus structural proteins: Promising candidates for antibody-based rapid diagnostic test development
Source: PLoS One. 2018 Dec 17;13(12):e0208851. doi: 10.1371/journal.pone.0208851 (PMC6296674; doi:10.1371/journal.pone.0208851)
Supplement: S3 Fig — (PDF) [file pone.0208851.s004.pdf]

S3 Fig

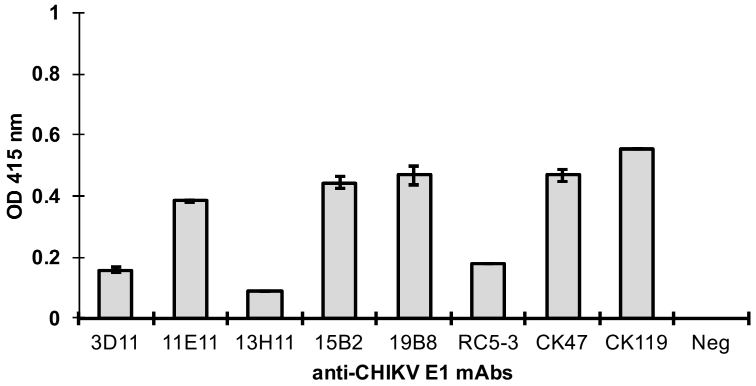

S3 Fig. Enzyme-Linked Immuno-Sorbent Assay (ELISA). We prepared the concentrated viral particles from CHIKV Asian genotype (strain ARUBA-1125)-containing culture supernatant by ultracentrifugation at 50,000 x g for 2.5 hours (Optima™ MAX-XP; Beckman-Coulter, IN, USA) and adjusted viral titers to 1 x 10^6 plaque forming units (PFU) /mL with 100 mM carbonate coating buffer, pH 9.6. The resulting viral particles were then coated into 96-well plate (100 µL per well) (NUNC-immuno plate, Thermo scientific, Roskilde, Denmark) overnight at 4 °C. After extensive wash with PBS containing 0.05% (v/v) Tween-20 (PBS-T) for 3 times, 100 µL of anti-CHIKV E1 (at final concentration of 2 µg/mL in PBS-T containing 1% (w/v) bovine serum albumin) as well as negative control (PBS) were then added into coated wells and incubated at 37 °C for 1 hour. Wells were then washed before adding 100 µL of alkaline phosphatase (ALP)-conjugated rabbit-anti-mouse IgG (Jackson ImmunoResearch, Inc.) in 1:2000 dilutions and incubated at 37 °C for 1 hour. After 3 times washing, 100 µL of 4-nitrophenyl phosphate disodium hexahydrate (Sigma-Aldrich, MU, USA) substrate at 1 mg/ml was added and incubated in the dark at room temperature for 30 minutes. Absorbance was read immediately at 415 nm using microplate reader (Sunrise Absorbance reader, Tecan group Ltd., Switzerland, with Magellan software version 7.1). Data was presented as optical density (OD). Six of newly produced anti-CHIKV E1 mAbs (3D11, 11E11, 13H11, 15B2, 19B8 and RC5-3) were used. Anti-CHIKV E1 mAbs (CK47 and CK119) and PBS were used as positive and negative controls, respectively. Gray bars represent the optical density (OD) at 415 nm. Error bars indicate actual fluctuations between measurements of two different wells.
