# Supplementary material for: Broad-spectrum monoclonal antibodies against chikungunya virus structural proteins: Promising candidates for antibody-based rapid diagnostic test development
Source: PLoS One. 2018 Dec 17;13(12):e0208851. doi: 10.1371/journal.pone.0208851 (PMC6296674; doi:10.1371/journal.pone.0208851)
Supplement: S4 Fig — (PDF) [file pone.0208851.s005.pdf]

S4 Fig

Chikungunya virus	Alexa Fluor 488	DAPI	Merge	mAb
CP10 strain	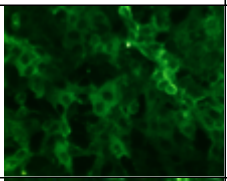	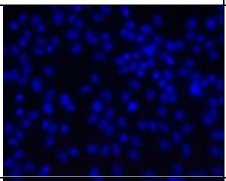	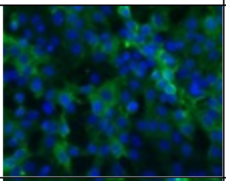	24B3
Ross low psg	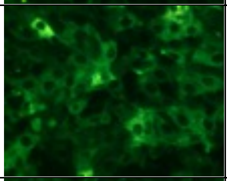	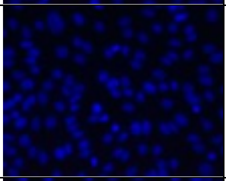	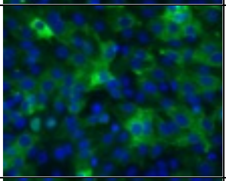	
A1125	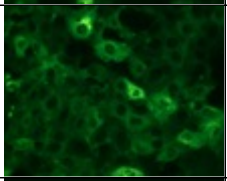	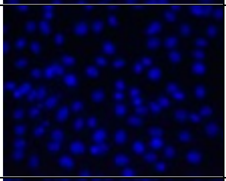	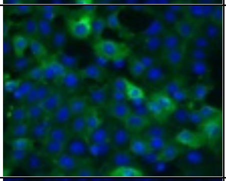	
Mock infection	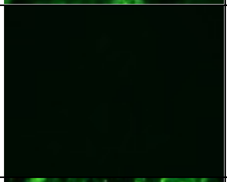	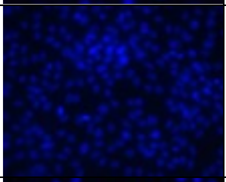	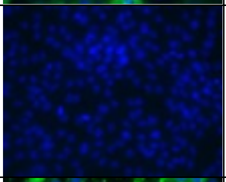	
CP10	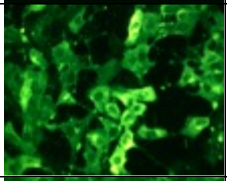	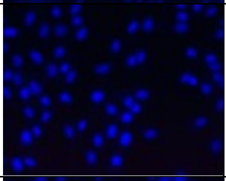	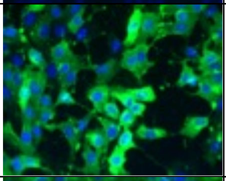	26A2
Ross low psg	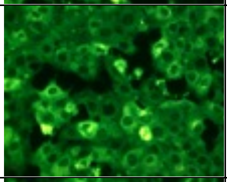	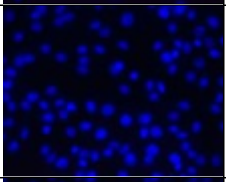	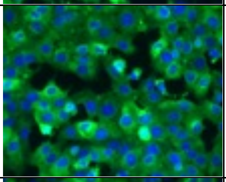	
A1125	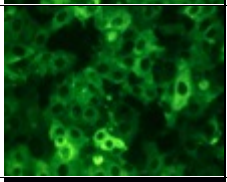	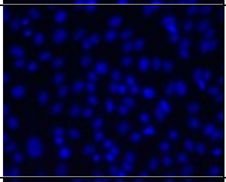	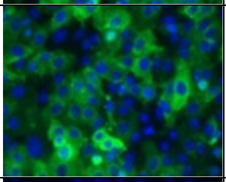	
Mock infection	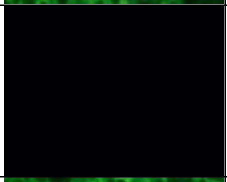	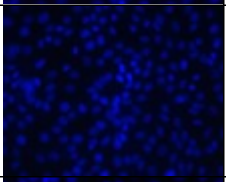	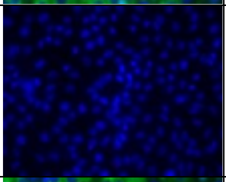	
CP10	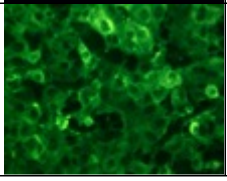	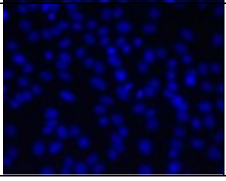	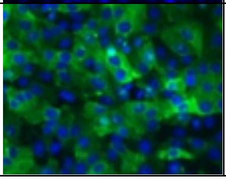	32A3

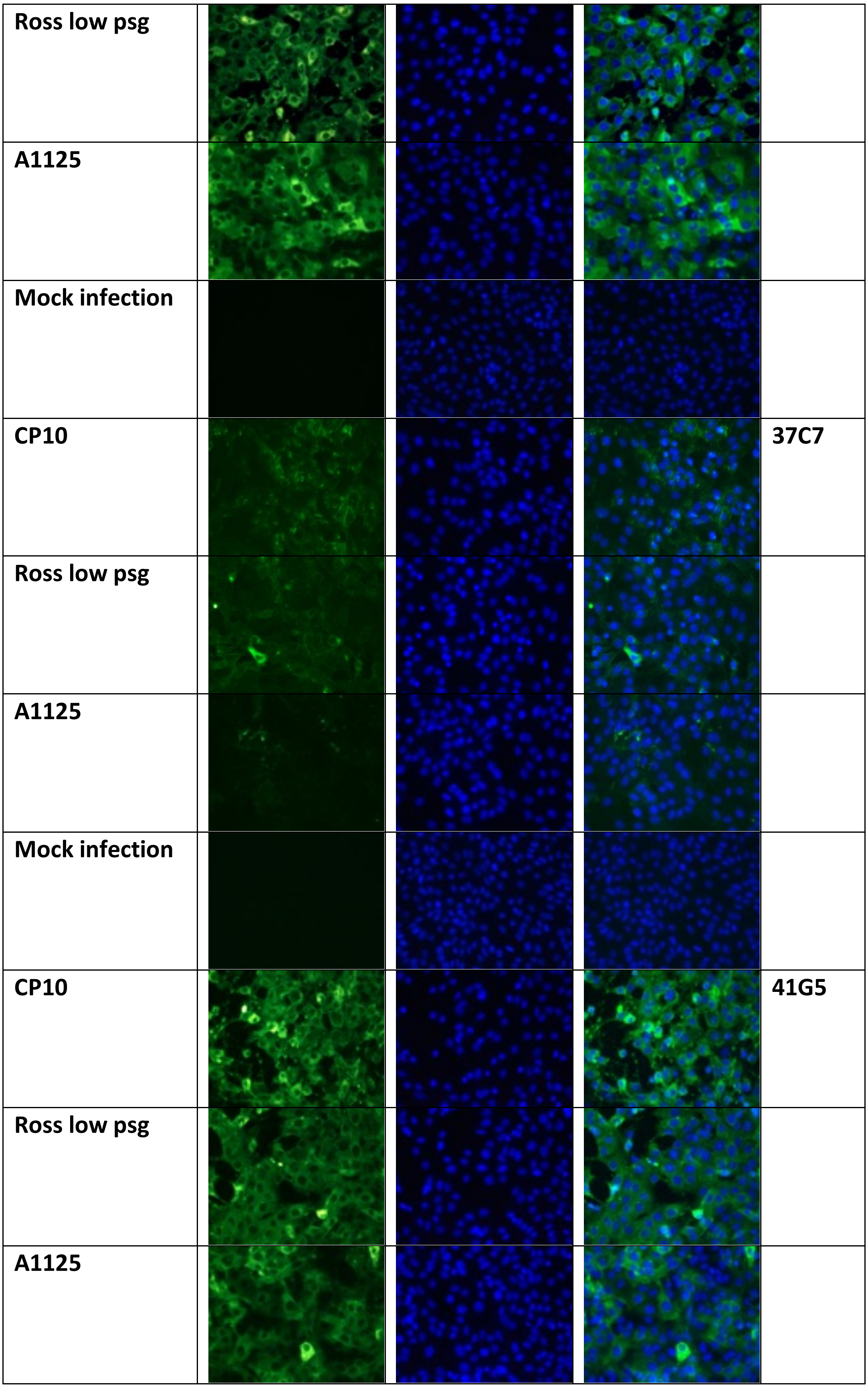
Ross low psg
A1125
Mock infection
CP10
37C7
Ross low psg
A1125
Mock infection
CP10
41G5
Ross low psg
A1125

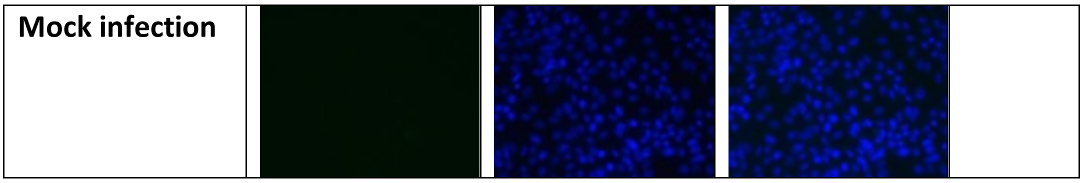

S4 Fig. Reactivity profile of anti-CHIKV capsid mAbs against CHIKV-infected Vero cells. Three strains of CHIKV (CP10 (ECSA-IOL), Ross low psg (ECSA genotype), and ARUBA-1125 (Asian genotype) were used to infect Vero cells. Infected cells were stained with anti-CHIKV E protein monoclonal antibodies (mAb), name as indicated. Mouse anti-CHIKV E1 mAbs CK47 (reactivity restricted to ECSA-IOL) and CK119 (broadly reactive to CHIKV) were used as positive controls to detect CHIKV virus-infected cells. The detection was based on Alexa Fluor 488-conjugated secondary antibody (green, left panels). DAPI nuclear counterstain was used to stain nuclei of cells (blue, middle panels). Alexa Fluor 488 and DAPI images were merged using ImageJ 1.5Oi (National Institutes of Health USA), and the merged images are shown in the right panels (Merge). Images are representative of results obtained from three independent experiments and were taken under 40x objective magnification using a fluorescence microscope (Carl Zeiss, Oberkochen, Germany).
