# Supplementary material for: Broad-spectrum monoclonal antibodies against chikungunya virus structural proteins: Promising candidates for antibody-based rapid diagnostic test development
Source: PLoS One. 2018 Dec 17;13(12):e0208851. doi: 10.1371/journal.pone.0208851 (PMC6296674; doi:10.1371/journal.pone.0208851)
Supplement: S6 Fig — (PDF) [file pone.0208851.s007.pdf]

S6 Fig

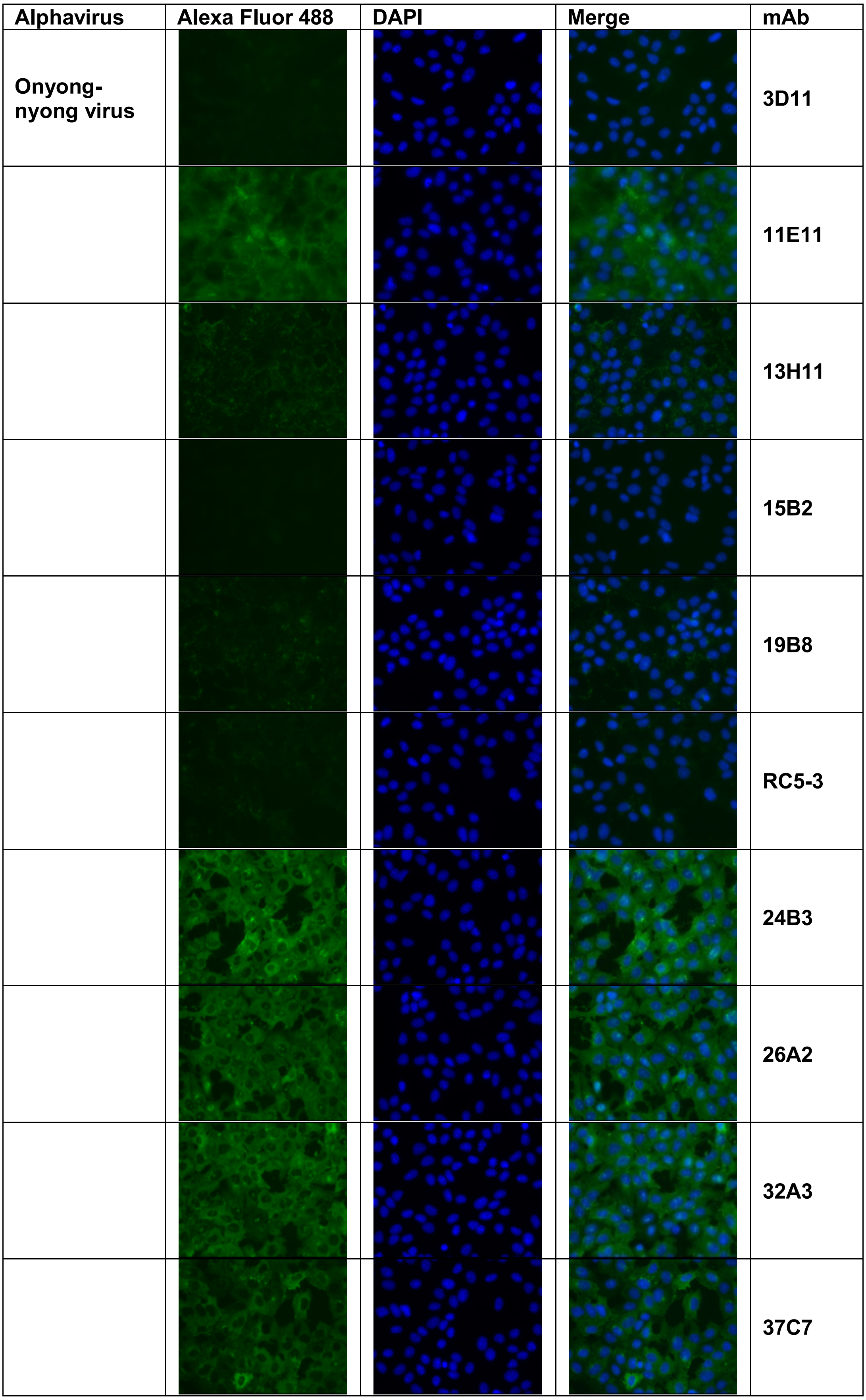
Alphavirus
Alexa Fluor 488
DAPI
Merge
mAb
Onyong-nyong virus
3D11
11E11
13H11
15B2
19B8
RC5-3
24B3
26A2
32A3
37C7

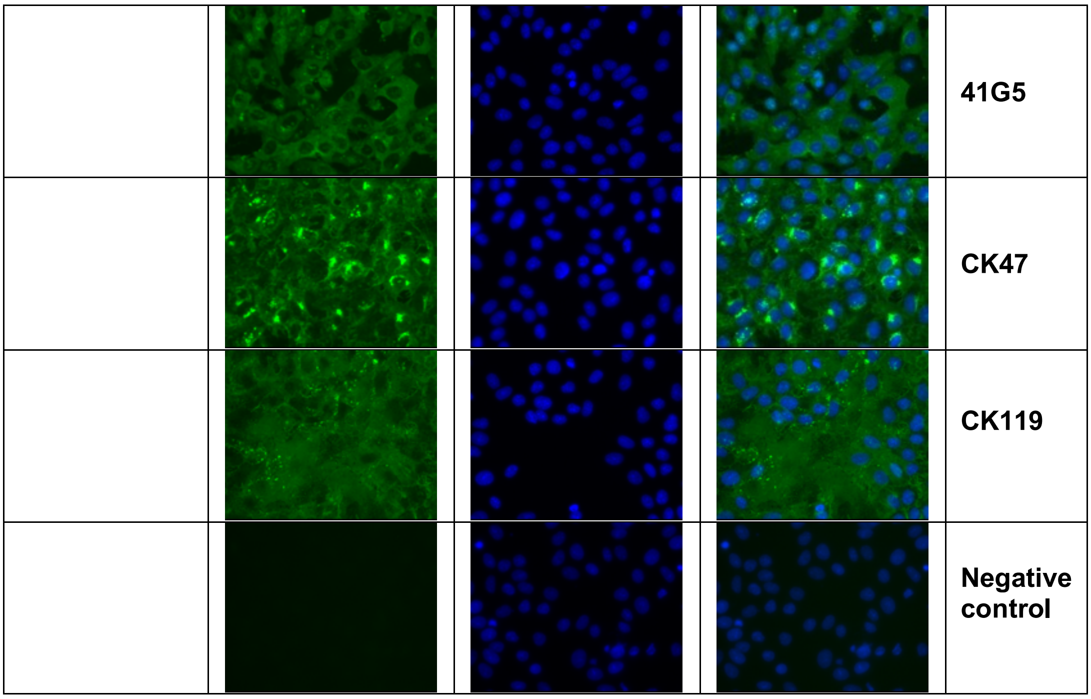

S6 Fig. Indirect immunofluorescence analysis of anti-CHIKV mAbs against O'nyong-nyong virus (ONNV) -infected Vero cells. ONNV (strain Ahero) was used to infect Vero cells. Infected cells were stained with anti-CHIKV E protein or capsid protein monoclonal antibodies (mAbs), name as indicated. The detection was based on Alexa Fluor 488-conjugated secondary antibody (green, left panels). DAPI nuclear counterstain was used to stain nuclei of cells (blue, middle panels). Alexa Fluor 488 and DAPI images were merged using MetaVue (Molecular Devices Japan), and the merged images are shown in the right panels (Merge). Images are representative of results obtained from two independent experiments and were taken under 40x objective magnification using a fluorescence microscope (ECLIPSE Ti2, Nikon, Tokyo, Japan).
