# Supplementary material for: Broad-spectrum monoclonal antibodies against chikungunya virus structural proteins: Promising candidates for antibody-based rapid diagnostic test development
Source: PLoS One. 2018 Dec 17;13(12):e0208851. doi: 10.1371/journal.pone.0208851 (PMC6296674; doi:10.1371/journal.pone.0208851)
Supplement: S9 Fig — (PDF) [file pone.0208851.s010.pdf]

S9 Fig

Alphavirus	Alexa Fluor 488	DAPI	Merge	mAb
Venezuelan Equine Encephalitis virus, strain 78v	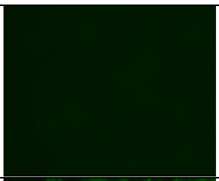	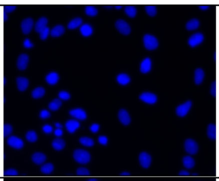	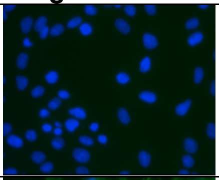	3D11
	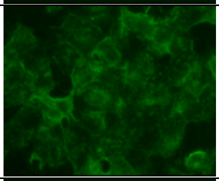	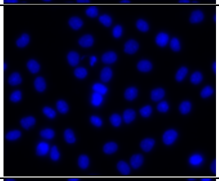	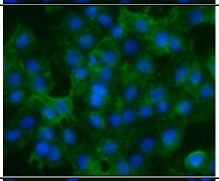	11E11
	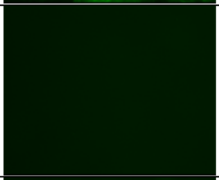	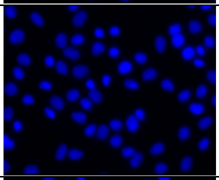	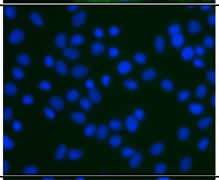	13H11
	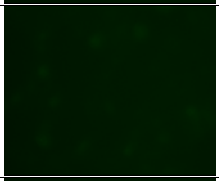	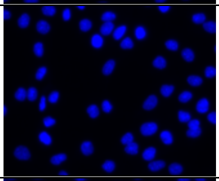	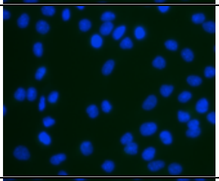	15B2
	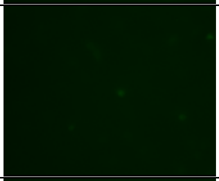	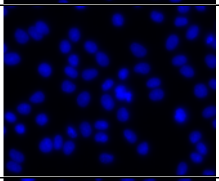	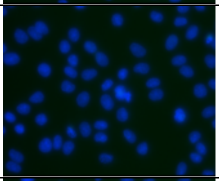	19B8
	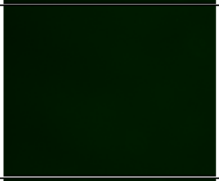	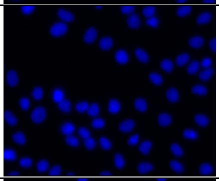	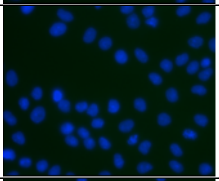	RC5-3
	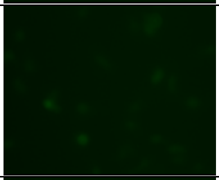	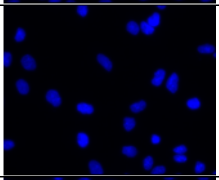	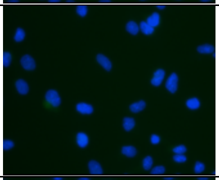	24B3
	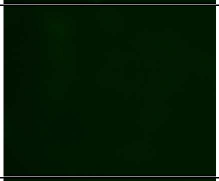	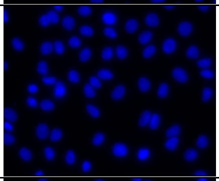	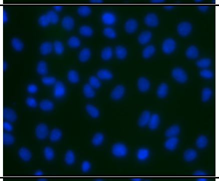	26A2
	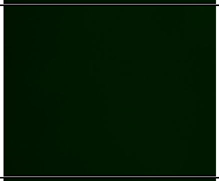	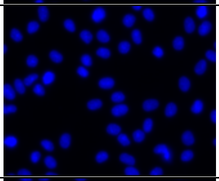	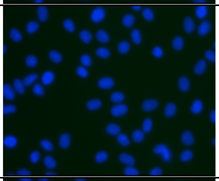	32A3
	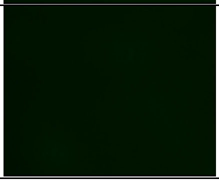	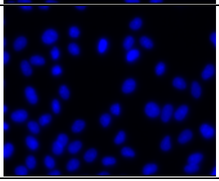	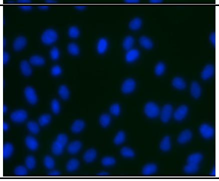	37C7

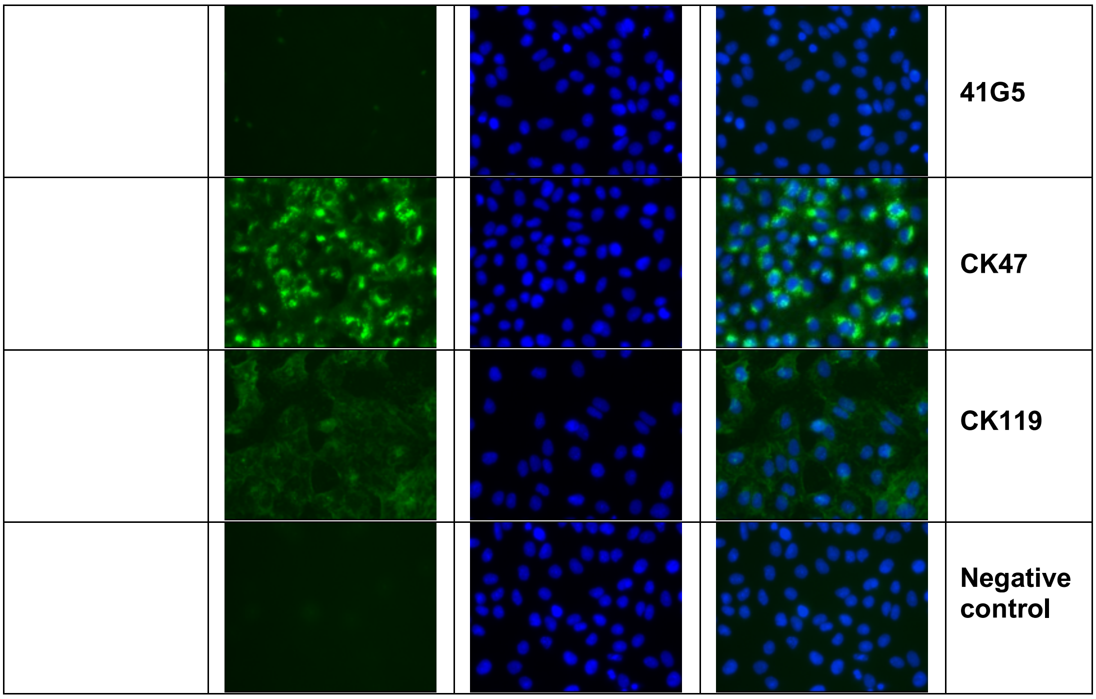

S9 Fig. Indirect immunofluorescence analysis of anti-CHIKV mAbs against Venezuelan Equine Encephalitis virus (VEEV) -infected Vero cells. VEEV (strain 78v) was used to infect Vero cells. Infected cells were stained with anti-CHIKV E protein or capsid protein monoclonal antibodies (mAbs), name as indicated. The detection was based on Alexa Fluor 488-conjugated secondary antibody (green, left panel). DAPI nuclear counterstain was used to stain nuclei of cells (blue, middle panels). Alexa Fluor 488 and DAPI images were merged using MetaVue (Molecular Devices Japan) and the merged images are shown in the right panels (Merge). Images are representative of results obtained from two independent experiments and were taken under 40x objective magnification using a fluorescence microscope (ECLIPSE Ti2, Nikon, Tokyo, Japan).
