# Supplementary material for: Broad-spectrum monoclonal antibodies against chikungunya virus structural proteins: Promising candidates for antibody-based rapid diagnostic test development
Source: PLoS One. 2018 Dec 17;13(12):e0208851. doi: 10.1371/journal.pone.0208851 (PMC6296674; doi:10.1371/journal.pone.0208851)
Supplement: S16 Fig — (PDF) [file pone.0208851.s017.pdf]

S16 Fig

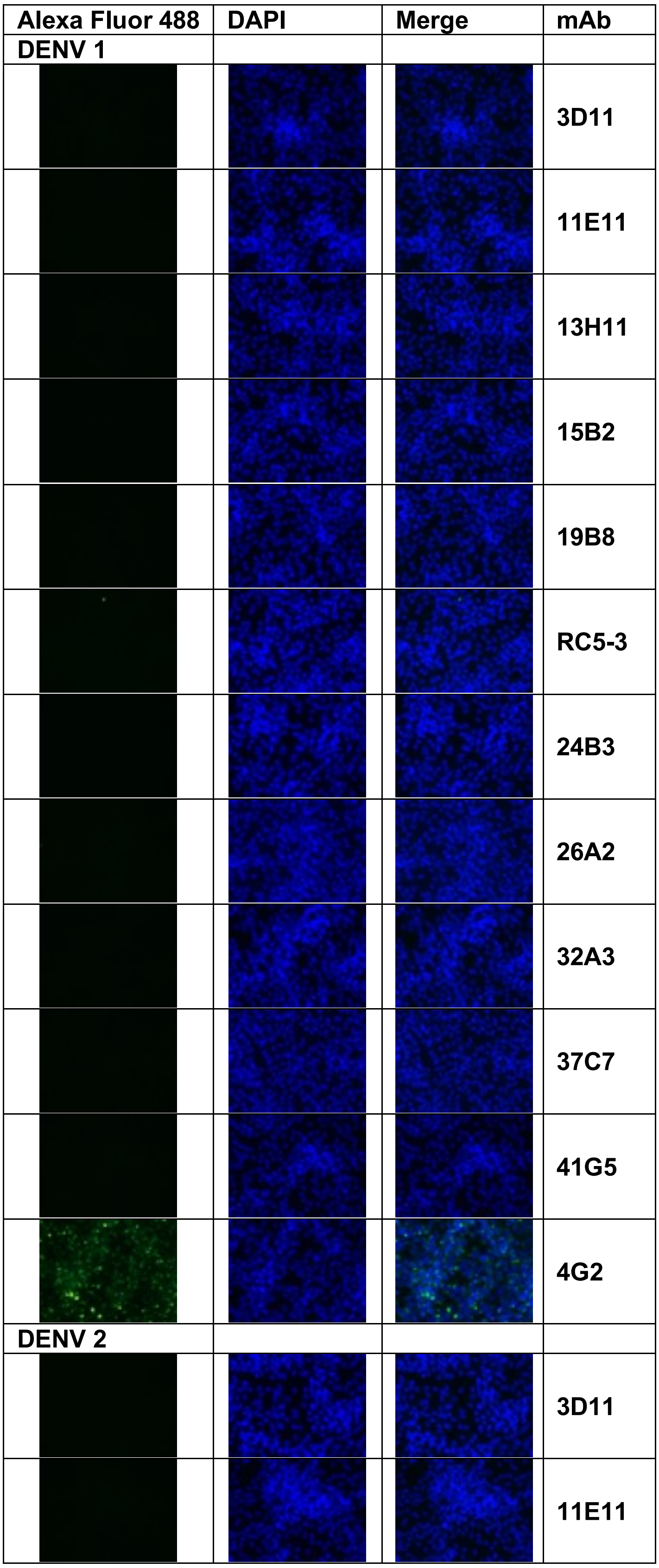

Alexa Fluor 488
DAPI
Merge
mAb
DENV 1
3D11
11E11
13H11
15B2
19B8
RC5-3
24B3
26A2
32A3
37C7
41G5
4G2
DENV 2
3D11
11E11

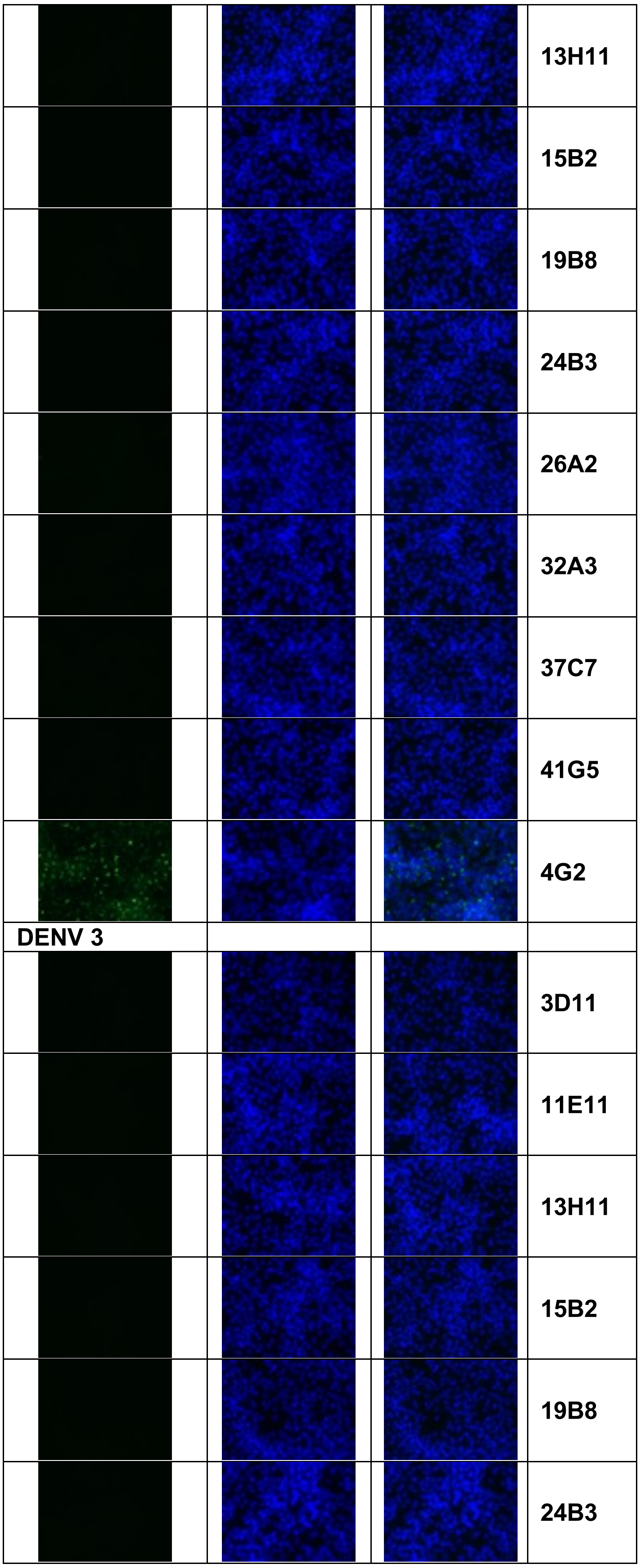
13H11
15B2
19B8
24B3
26A2
32A3
37C7
41G5
4G2
DENV 3
3D11
11E11
13H11
15B2
19B8
24B3

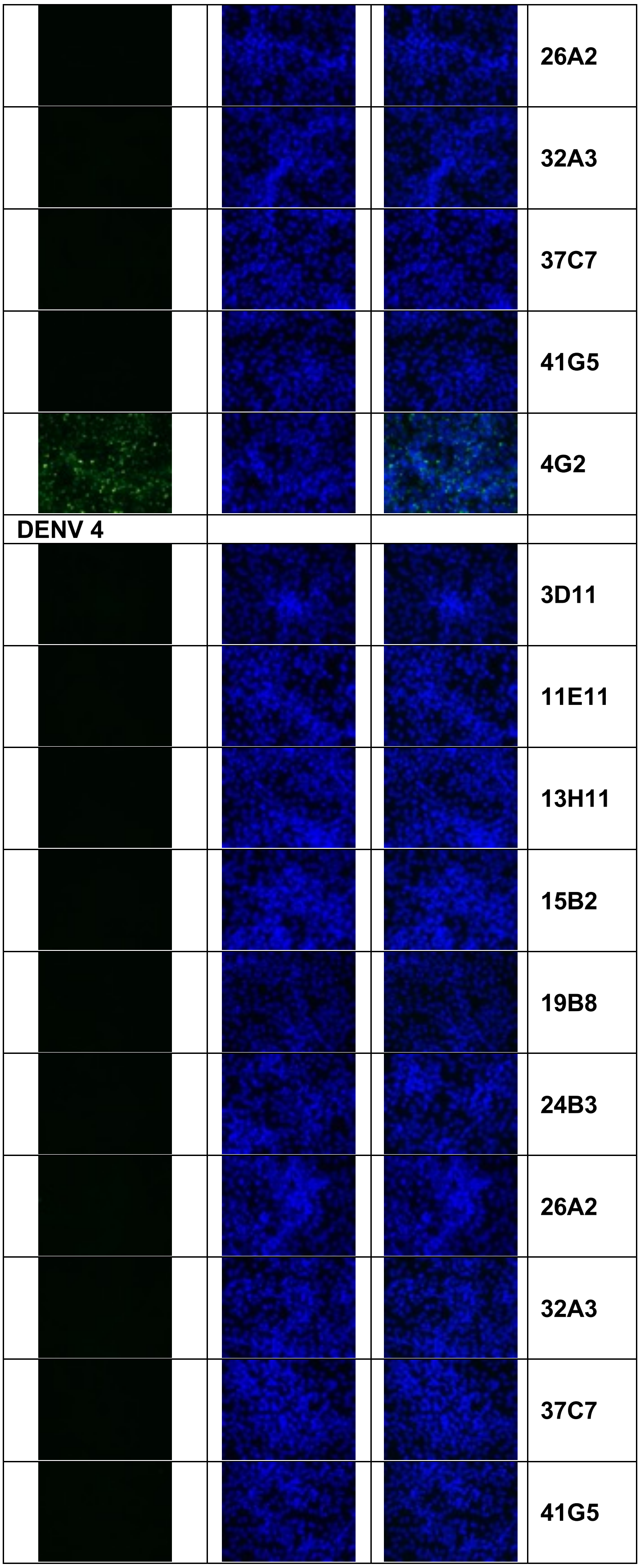
26A2
32A3
37C7
41G5
4G2
DENV 4
3D11
11E11
13H11
15B2
19B8
24B3
26A2
32A3
37C7
41G5

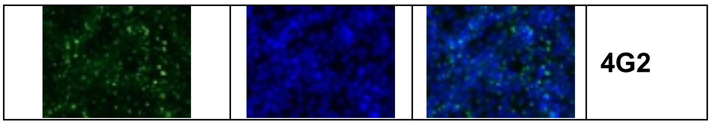

S16 Fig. Indirect immunofluorescence analysis of anti-CHIKV mAbs against Dengue virus-infected Vero cells. Four serotypes of Dengue virus (DENV; using strains Mochizuki, 16681, H87, and H241 as representatives of the DENV1 to DENV4 serotypes, respectively) were used to infect Vero cells. Infected cells were stained with anti-CHIKV E protein or capsid protein monoclonal antibodies (mAbs), name as indicated. Mouse anti-flavivirus mAb 4G2 was used as a positive control to detect Dengue virus-infected cells. The detection was based on Alexa Fluor 488-conjugated secondary antibody (green, left panels). DAPI nuclear counterstain was used to stain nuclei of cells (blue, middle panels). Alexa Fluor 488 and DAPI images were merged using ImageJ 1.5Oi (National Institutes of Health USA), and the merged images are shown in the right panels (Merge). Images are representative of results obtained from two independent experiments and were taken under 40x objective magnification using a fluorescence microscope (Carl Zeiss, Oberkochen, Germany).
